# Structural insights into ligand recognition and selectivity of somatostatin receptors

**DOI:** 10.1038/s41422-022-00679-x

**Published:** 2022-06-23

**Authors:** Wenli Zhao, Shuo Han, Na Qiu, Wenbo Feng, Mengjie Lu, Wenru Zhang, Mu Wang, Qingtong Zhou, Shutian Chen, Wei Xu, Juan Du, Xiaojing Chu, Cuiying Yi, Antao Dai, Liaoyuan Hu, Michelle Y. Shen, Yaping Sun, Qing Zhang, Yingli Ma, Wenge Zhong, Dehua Yang, Ming-Wei Wang, Beili Wu, Qiang Zhao

**Affiliations:** 1 State Key Laboratory of Drug Research and CAS Key Laboratory of Receptor Research, Shanghai Institute of Materia Medica, Chinese Academy of Sciences, Shanghai, China; 2 University of Chinese Academy of Sciences, Beijing, China; 3 School of Pharmaceutical Science and Technology, Hangzhou Institute for Advanced Study, UCAS, Hangzhou, Zhejiang China; 4 Department of Pharmacology, School of Basic Medical Sciences, Fudan University, Shanghai, China; 5 School of Chinese Materia Medica, Nanjing University of Chinese Medicine, Nanjing, Jiangsu China; 6 School of Life Science and Technology, ShanghaiTech University, Shanghai, China; 7 The National Center for Drug Screening, Shanghai Institute of Materia Medica, Chinese Academy of Sciences, Shanghai, China; 8 Amgen Asia R&D Center, Shanghai, China; 9 Regor Therapeutics, Shanghai, China; 10 Zhongshan Institute of Drug Discovery, Shanghai Institute of Materia Medica, Chinese Academy of Sciences, Zhongshan, Guangdong China

**Keywords:** Cryoelectron microscopy, X-ray crystallography

## Abstract

Somatostatin receptors (SSTRs) play versatile roles in inhibiting the secretion of multiple hormones such as growth hormone and thyroid-stimulating hormone, and thus are considered as targets for treating multiple tumors. Despite great progress made in therapeutic development against this diverse receptor family, drugs that target SSTRs still show limited efficacy with preferential binding affinity and conspicuous side-effects. Here, we report five structures of SSTR2 and SSTR4 in different states, including two crystal structures of SSTR2 in complex with a selective peptide antagonist and a non-peptide agonist, respectively, a cryo-electron microscopy (cryo-EM) structure of G_i1_-bound SSTR2 in the presence of the endogenous ligand SST-14, as well as two cryo-EM structures of G_i1_-bound SSTR4 in complex with SST-14 and a small-molecule agonist J-2156, respectively. By comparison of the SSTR structures in different states, molecular mechanisms of agonism and antagonism were illustrated. Together with computational and functional analyses, the key determinants responsible for ligand recognition and selectivity of different SSTR subtypes and multiform binding modes of peptide and non-peptide ligands were identified. Insights gained in this study will help uncover ligand selectivity of various SSTRs and accelerate the development of new molecules with better efficacy by targeting SSTRs.

## Introduction

Somatostatin (SST), an inhibitory hormone that distributes widely in both human central nervous system and periphery tissues negatively regulates multiple hormone releases (growth hormone, glucagon, insulin, gastrin and cholecystokinin) and cell proliferation through SST receptors (SSTRs).^1–4^ There are five types of SSTRs, namely SSTR1–SSTR5, which are divided into two subfamilies: SRIF1 (SSTR2, SSTR3, SSTR5) and SRIF2 (SSTR1, SSTR4) based on phylogenetic, sequence homology and ligand binding profiles.^5^ Two bioactive SST peptides, SST-14 and its N-terminally extended form SST-28, have been identified in mammals and show high and equal affinity for SSTR1–5.^6^ Among all the SSTRs, SSTR2 is the best characterized member with multiple effects on hormone secretion, cell cycling, apoptosis and angiogenesis.^7^ It is also the most common subtype expressed in both human neuroendocrine tumors (NETs) and related hormone diseases, making it a valuable target for diagnosis and therapy of tumors as well as acromegaly.^8,9^ On the contrary, SSTR4 is highly expressed in the central nervous system and mediates potent analgesic and anti-inflammatory actions.^10^ In addition, studies in recent years have revealed that SSTR4 agonists represent a promise for non-opioid pain control, especially for chronic neuropathic, inflammatory and mixed pain.^10,11^

Along with increased knowledge of the pharmacological effects of these receptors, medical application of SST and its analogs has expanded. A considerable amount of effort has been made to develop new therapeutics for oncology of SSTRs. Due to a very short half-life of SST (less than 3 min), several of its agonist analogs, especially octreotide and lanreotide, are used to treat acromegaly and NETs by targeting SSTR2, while its antagonist analog, CYN 154806, has been employed to study diverse functions of this receptor.^12^ It was found recently that radiolabeled SSTR antagonists produced superior images than that of agonists, pointing to a potential application in imaging and treating SSTR-expressing tumors.^13,14^ However, there are concerns about limited effectiveness and adverse events such as gastrointestinal disturbance and hyperglycemia.^15^ Since peptides have relatively short half-life and poor penetration to the blood–brain barrier, non-peptide ligands with high potency and subtype selectivity have been developed for each SSTR subtype showing different pharmacological properties.^5^ L-054,522 was identified and optimized by Merck that mimics the side chains of W8 and K9 at the β turn tip of the endogenous peptide SST-14, and displayed at least 3000-fold better selectivity for SSTR2.^16,17^ This full agonist exerts an inhibitory effect on growth hormone and glucagon releases, while SSTR4 selective agonist, J-2156, mediates pain relief.^18^

To reveal ligand selectivity and activation mechanisms of SSTRs, we solved the crystal structures of SSTR2 bound to selective peptide antagonist CYN 154806 and non-peptide agonist L-054,522, as well as cryo-EM complex structures of SSTR2–G_i1_ bound to endogenous ligand SST-14, SSTR4–G_i1_ bound to SST-14 and SSTR4–G_i1_ bound to non-peptide agonist J-2156, respectively. Combined with mutagenesis, molecular docking and molecular dynamics (MD) simulation studies, these structures reveal the key signature shared by their ligands which is prerequisite for receptor binding. Our findings also provide molecular insights into ligand selectivity, receptor activation and G protein coupling thereby offering near-atomic-resolution models for rational design of better drugs against SSTRs.

## Results

### Structure determination of SSTR2 and SSTR4 complexes

To facilitate the lipidic cubic phase (LCP) crystallization of SSTR2 with its peptide antagonist CYN 154806, the flexible C-terminus was truncated to T359 and bacillus subtilis xylanase was inserted between S238 and G243 of the intracellular loop 3 (ICL3). Three mutations, D89^2.50^N, V106^ECL1^E and S316^8.47^D (superscript numbers represent Ballesteros–Weinstein nomenclature^19^), were introduced to improve protein yield and homogeneity. To solve the crystal structure of L-054,522-bound SSTR2, D89^2.50^ was reinstated as in wild type (WT) and the junction site of xylanase was adjusted between I240 and V242 to improve crystal quality. Crystals of both complexes were obtained in monoolein lipid phases and determined at 2.65 Å and 2.6 Å resolution, respectively (Supplementary information, Table S1). These modifications had decreased the ligand binding for CYN 154806 by ~50-fold compared with the WT but had little effect on L-054,522 binding and signaling (Supplementary information, Fig. S1a, b and Tables S3 and S4).

For cryo-EM studies, SSTR2 WT with C-terminal truncation to T359 and SSTR4 with C-terminal truncation to L328 plus a V264^6.40^F mutation were prepared to facilitate complex formation. Binding and signaling assays showed that these modifications had no influence on receptor activities (Supplementary information, Fig. S1a–d and Tables S3 and S4).^20^ To obtain stable complexes, three subunits of G_i1_ protein were co-expressed with the receptors in High-Five insect cells. Complexes were assembled in the membrane and a single-chain variable Fab fragment (scFv16) was applied to stabilize the SSTR4–G_i1_ complexes.^21^ Structures were determined by single-particle cryo-EM at a nominal resolution of 3.1 Å (SST-14–SSTR2–G_i1_), 2.9 Å (SST-14–SSTR4–G_i1_) and 2.8 Å (J-2156–SSTR4–G_i1_), respectively (Supplementary information, Figs. S2, S3 and Table S2).

**Fig. 1:**
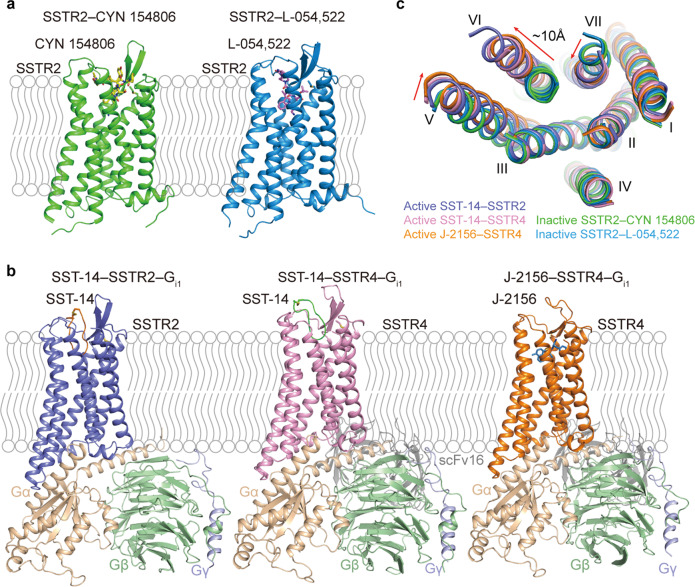
Overall structures of SSTR2 and SSTR4 complexes. **a** Crystal structures of SSTR2 in complex with CYN 154806 and L-054,522, respectively. SSTR2 is shown as cartoon and colored by green and protactinium separately. CYN 154806 and L-054,522 are shown as sticks and colored by yellow and violet, respectively. Disulfide bonds are shown as yellow sticks. **b** Cryo-EM structures of SST-14–SSTR2–G_i_, SST-14–SSTR4–G_i_ and J-2156–SSTR4–G_i_ complexes. SSTR2 is shown as cartoon and colored by slate. SSTR4 is shown as cartoon and colored by pink and orange. Gα_i1_, Gβ, Gγ and scFv16 are colored by wheat, pale green, light blue and gray. SST-14 is shown as cartoon and colored by orange and green in SSTR2–G_i_ and SSTR4–G_i_ complexes, respectively. J-2156 is shown as sky blue sticks. **c** Intracellular view of structural comparison of solved structures. Red arrows indicate the movements of helices V, VI and VII.

The overall structures of SSTR2 and SSTR4 possess the canonical seven-transmembrane (7-TM) architecture with an extended helix VIII in parallel with the membrane (Fig. 1a, b). Similar to other solved peptide-bound class A G protein-coupled receptors (GPCRs), extracellular loop 2 (ECL2) of SSTR2 and SSTR4 form short antiparallel β-strands stabilized by conserved disulfide bonds between Cys^3.25^ and Cys^ECL2^ (Fig. 1a, b). Among the five structures, two crystal structures are in inactive state or in agonist-bound inactive state, probably due to the fact that conformations with low energy states facilitated the crystallization (Fig. 1c). In contrast, the G_i1_-coupled SSTR2 and SSTR4 adopt full active states with a remarkable outward displacement of helix VI (~10 Å, measured by Cα of 6.29) accompanied by transverse movement of helix V and inward movement of helix VII, which are consistent with activation characteristics of class A GPCRs (Fig. 1c).^22,23^ In spite of binding to G_i1_ and different ligands, SSTR2 and SSTR4 structurally resemble each other with root-mean-square-deviation (RMSD) values of 1.3–1.5 Å for the Cα atoms (Supplementary information, Fig. S4a). However, G protein binding between the two receptors still exhibit several conformational differences. Compared to the SST-14–SSTR4–G_i1_, the SST-14–SSTR2–G_i1_ complex shows that the C-terminus of α5 helix of Gα_i1_ tilts ~2 Å toward helix VI (measured by the Cα of F354 of Gα_i1_), which further induces an outward movement of helix VI but inward movement of ICL3 of SSTR2 as opposed to SSTR4 (Supplementary information, Fig. S4b). It appears that diverse residues of ICL3 between SSTR2 and SSTR4 form different interactions with Gα_i1_. S244^ICL3^ of SSTR2 makes polar interactions with the side chain of E318 of Gα_i1_; however, W247^ICL3^, its counterpart in SSTR4, pushes the ICL3 away due to a bulky side chain (Supplementary information, Fig. S4c). The ICL3 in closer proximity forms further interactions between SSTR2 and Gα_i1_, e.g., K246^ICL3^ makes a hydrogen bond with the main chain of D315 of Gα_i1_, which is not observed in SSTR4 (Supplementary information, Fig. S4c). The closer contacts lead to a larger interaction area between SSTR2 and Gα_i1_ (~1129 Å^2^) than that between SSTR4 and Gα_i1_ (~965 Å^2^), thereby contributing to a stronger binding of Gα_i1_ towards SSTR2. Indeed, according to our binding and signaling data, even though SST-14 binds to both receptors with similar affinities (1.4 nM vs 1.5 nM), it displays 5-fold higher potency in activating SSTR2 compared to SSTR4 (Supplementary information, Fig. S1, Tables S3 and S4).

### Peptide binding to SSTR2 and SSTR4

**Fig. 2:**
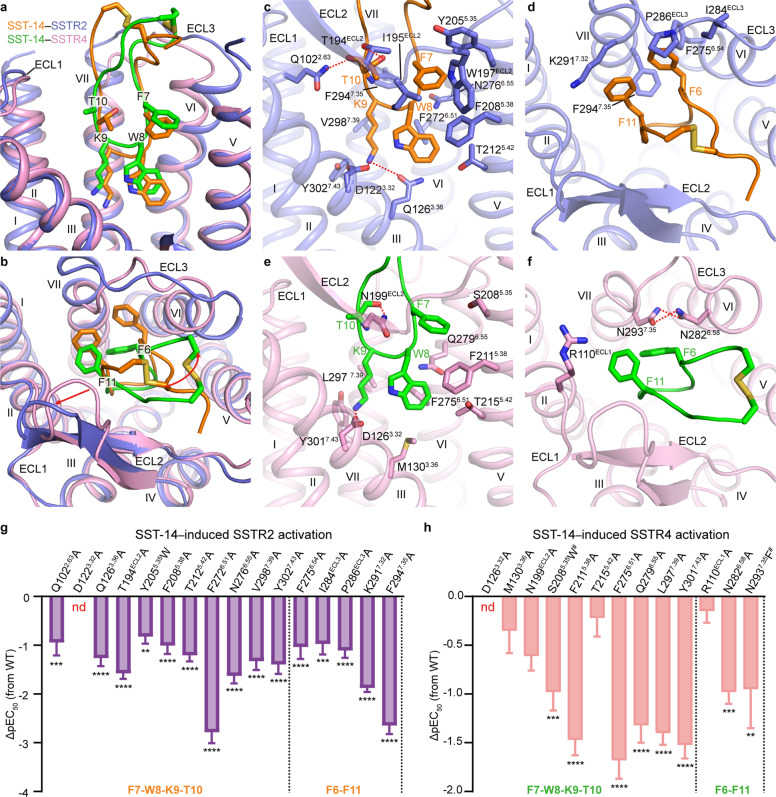
Binding modes of SST-14 in SSTR2 and SSTR4. **a** Side view of superimposition of SST-14-bound SSTR2 and SSTR4. SSTR2 and SSTR4 are shown as cartoon and colored in slate and pink, respectively. SST-14 is shown as cartoon and colored in orange and green, respectively. Disulfide bonds of SST-14 are shown as yellow sticks. **b** Extracellular view of structural superimposition of SST-14-bound SSTR2 and SSTR4. The rotation of the disulfide bond is indicated by red arrow. **c** Detailed interactions between the key pharmacophore (F7–W8–K9–T10) of SST-14 and SSTR2. Residues of SSTR2 are shown as slate sticks. Residues of SST-14 are shown as orange sticks. Polar interactions are indicated by red dash lines. **d** Detailed interactions between F6 and F11 of SST-14 and SSTR2. **e** Detailed interactions between the key pharmacophore (F7-W8-K9-T10) of SST-14 and SSTR4. Residues of SSTR4 are shown as pink sticks. Residues of SST-14 are shown as green sticks. **f** Detailed interactions between F6 and F11 of SST-14 and SSTR4. **g**, **h** Inhibition of forskolin-stimulated cAMP accumulation of WT SSTR2 and SSTR2 mutants (**g**) or WT SSTR4 and SSTR4 mutants (**h**) induced by SST-14 using HEK293F cells. The mutants are divided into two groups by dashed lines: (i) mutations of the residues that interact with the key pharmacophore (F7–W8–K9–T10) of SST-14; (ii) mutations of the residues that interact with F6 and F11 of SST-14. Bars represent the differences between the calculated SST-14 potency (pEC_50_) of WT and mutants. Data are shown as means ± SEM from at least three independent experiments. One-way ANOVA was performed followed by Dunnett’s post-test and compared with WT. The *P* value was defined as: *P < 0.05; **P < 0.01; ***P < 0.001; ****P < 0.0001. nd (not determined) indicates that a robust concentration response curve could not be determined within the concentration range tested. ^#^Low surface expression level (< 40% of WT expression). Detailed statistical evaluation is shown in Supplementary information, Table S3.

SST-14 (A1–G2–C3–K4–N5–F6–F7–W8–K9–T10–F11–T12–S13–C14) is a cyclic tetra-decapeptide with a disulfide bond between C3 and C14.^24^ To accommodate the cyclic SST-14, the extracellular part of SSTR2 and SSTR4 are widely opened like other peptide receptors that bind to cyclic peptide ligands (Supplementary information, Fig. S5a).^25–29^ The disulfide bonds in these cyclic peptides orientate to different directions upon binding to different receptors, revealing the divergent binding modes of cyclic peptides for peptide receptors (Supplementary information, Fig. S5a). For SST-14, it adopts a disulfide-stabilized β hairpin structure with the key pharmacophore (F7–W8–K9–T10) at the tip of the turn. The tip of the hairpin penetrates into the widely opened helical core with the carboxyl and amino termini and the disulfide bond exposed to the extracellular milieu in both receptors (Fig. 2a, b). The conformations of SST-14 outside the pharmacophore are somewhat different between the two receptors, as the disulfide bond of SST-14 in SSTR2 rotates clockwise to ECL2 and pulls ECL2 outward compared with SST-14 in SSTR4 (Fig. 2b). Of interest is that albeit bound to the same peptide, the receptor–ligand interaction area of SST-14–SSTR2 (~1140 Å^2^) is ~300 Å^2^ larger than that of SST-14–SSTR4 (~836 Å^2^), which might be caused by the different binding conformations of SST-14 in different receptors (Supplementary information, Fig. S5b).

It has been shown that the W8–K9 residue pair existing at the β turn tip of SST is essential for bioactivities, and indeed it forms the tip of β hairpin and sits at the bottom of the binding pocket in both receptors (Fig. 2c, e). In SSTR2, the protonated nitrogen atom of K9 forms a salt bridge with D122^3.32^ and a hydrogen bond with Q126^3.36^, which function as anchors at the bottom of the binding pocket (Fig. 2c). D122^3.32^A and Q126^3.36^A mutations dramatically impaired the signaling and ligand binding of SST-14 to SSTR2 (Fig. 2g; Supplementary information, Tables S3 and S4), indicating that these residues play a central role in ligand recognition. The alkyl chain of K9 is also involved in the hydrophobic core formed by F272^6.51^, V298^7.39^ and Y302^7.43^ and the indole group of W8 interacts with F208^5.38^, T212^5.42^, F272^6.51^ and N276^6.55^ (Fig. 2c). Alanine mutations of these residues impaired SST-14-induced receptor activation (Fig. 2g). Among them, mutation of F272^6.51^ which forms the strongest interaction with SST-14, showed ~600-fold reduction in SST-14-induced G_i_ signaling (Fig. 2g; Supplementary information, Table S3). The side chains of F7 and T10 of SST-14 adopt similar orientations and extend toward helix V and ECL2 of SSTR2. F7 is encompassed by the hydrophobic pocket formed by ECL2 and helix V, including I195^ECL2^, W197^ECL2^, Y205^5.35^ and F208^5.38^, and T10 is anchored by hydrogen bonds with Q102^2.63^ and T194^ECL2^ (Fig. 2c). Replacement of F7 by mesityl-alanine might cause spatial clash to this hydrophobic pocket and significantly affect binding of SST-14 to SSTR2.^30^ Mutants such as Y205^5.35^W which blocked the hydrophobic pocket also decreased the potency of SST-14 as well (Fig. 2g; Supplementary information, Table S3). Apart from the hydrophobic pocket, ECL2 is equally important for SST-14 recognition. Alanine mutation of T194^ECL2^ compromised the potency of SST-14 by ~40-fold (Fig. 2g; Supplementary information, Table S3). Previous studies demonstrated that rather than interacting with the receptor, two aromatic side chains of F6 and F11 contact with each other and stabilize the active conformation of SST-14.^31^ However, in our SST-14–SSTR2 complex structure, both F6 and F11 make strong hydrophobic interactions with SSTR2 (Fig. 2d). F6 of SST-14 in SSTR2 is stabilized by a hydrophobic cavity formed by F275^6.54^, I284^ECL3^, P286^ECL3^ and F294^7.35^ of helix VI, ECL3 and helix VII, while F11 is stabilized by hydrophobic interactions with F6 of SST-14 and the alkyl chain of K291^7.32^ (Fig. 2d). Disruption of the hydrophobic network between F6 and F11 weakened SST-14 potency, especially for F294^7.35^A, which decreased SST-14 potency by > 200-fold (Fig. 2g; Supplementary information, Table S3).

In SSTR4, SST-14 occupies a similar binding pocket as in SSTR2 with the key pharmacophore (F7–W8–K9–T10) located at the bottom of the pocket to form conserved interactions (Fig. 2a, e). However, due to the conformational difference between SSTR2 and SSTR4, the residues outside the pharmacophore rotate counterclockwise by ~30 degrees with its disulfide twisting ~5 Å more toward helices V and VI (Fig. 2b). Upon binding to different SSTRs, a certain level of flexibility is required for peptides. As a result of peptide rotation and twist, the main chain of T10 of SST-14 in SSTR4 is flipped to the opposite side compared to that in SSTR2 due to the difference in their overall conformation (Fig. 2e). Thus, the interaction between the side chain of T10 and ECL2 in SSTR2 is missing in SSTR4 because of the side chain flip. However, this is somewhat compensated by the hydrogen bond between non-conserved N199^ECL2^ of SSTR4 and the main chain of SST-14 (Fig. 2e). In addition, the conformational flexibility of SST-14 enables the side chains of F6 and F11 to adopt totally different orientations in SSTR4. The side chain of F6 bends toward helix II and only forms hydrophobic interactions with the side chain of N293^7.35^ in SSTR4 (Fig. 2f). The conformation of F6 side chain is further stabilized by the face-to-edge interaction with F11 of SST-14 (Fig. 2f). This could be explained by the fact that both ECL3 and helix VII are shorter by two residues in SRIF2 subfamily compared to those in SRIF1 and thus the hydrophobic pocket constituted by helix VI, helix VII and ECL3 in SSTR2 is replaced by a smaller pocket with more polar environment in SSTR4 which failed to accommodate the hydrophobic phenyl ring of F6 (Fig. 2f; Supplementary information, Fig. S6). N293^7.35^ and N282^6.58^ of SSTR4 form two hydrogen bonds and lock helices VI and VII in a closer conformation (Fig. 2f). Breaking these interactions by mutations such as N282^6.58^A decreased the EC_50_ of SST-14 by ~10-fold (Fig. 2h; Supplementary information, Table S3).

**Fig. 3:**
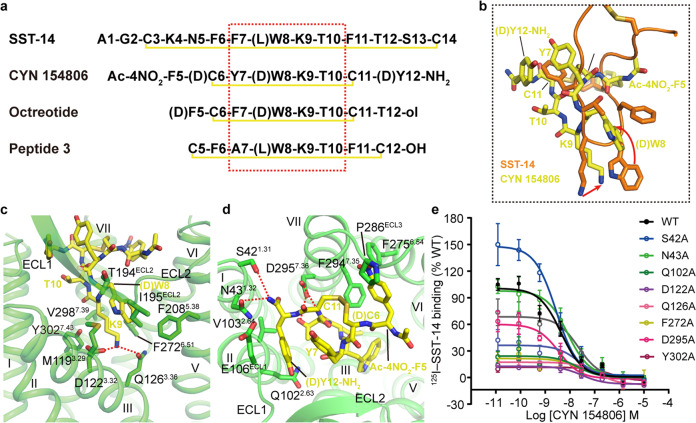
Binding mode of peptide antagonist CYN 154806. **a** Sequence alignment of SST-14, CYN 154806, octreotide and peptide 3. Key pharmacophore (F7–W8–K9–T10) is indicated by red dotted box; disulfide bonds are indicated by yellow lines. Sequence numbers are labeled based on SST-14. **b** Comparison of the binding modes of CYN 154806 and SST-14 in SSTR2. CYN 154806 is shown as yellow sticks and SST-14 is shown as cartoon and sticks in orange. Disulfide bonds are shown as yellow sticks. Conformational changes are indicated by red arrows. **c** Interactions between the d-W8–K9 of CYN 154806 and SSTR2. Residues involved in ligand binding are shown as green sticks. Polar interactions are indicated by red dash lines. **d** Interactions between the outside of d-W8–K9 of CYN 154806 and SSTR2. **e** Ligand binding of WT SSTR2 and SSTR2 mutants in the residues that interact with CYN 154806 in competition with ^125^I-SST-14. All data are shown as means ± SEM from at least three independent experiments performed in triplicate. Detailed statistical evaluation is shown in Supplementary information, Table S4.

CYN 154806 (Ac-NO_2_-F5–d-C6–Y7–d-W8–K9–T10–C11–Y12-NH2, disulfide bridge: d-C6–C11, numbered as in SST-14) is a selective antagonist of SSTR2 and shares the same backbone with the octapeptide agonist octreotide (d-F5–C6–F7–d-W8–K9–T10–C11–T12-ol) with inverted chirality of the fifth and sixth residues (Fig. 3a).^32^ However, CYN 154806 is an antagonist of SSTR2 while octreotide retains the ability to activate this receptor. To reveal inhibition and activation mechanisms of different peptide ligands, we docked octreotide into the SSTR2 model and found that it appears to adopt a conformation similar to SST-14. Structure superimposition with SST-14-bound SSTR2 indicates that CYN 154806 occupies a similar binding pocket to SST-14 in SSTR2 with different binding modes (Fig. 3b). K9 of CYN 154806 anchored in a similar environment and forms a salt bridge with D122^3.32^, a hydrogen bond with Q126^3.36^ and multiple hydrophobic interactions with F272^6.51^, V298^7.39^ and Y302^7.43^, as confirmed by our mutagenesis data (Fig. 3c, e; Supplementary information, Table S4). However, the main chain of d-W8–K9 pair in CYN 154806 rotates ~70 degrees counterclockwise around the Cα of K9 in comparison with SST-14, which may lead to an antagonist effect of CYN 154806 (Fig. 3b). In terms of the conformational rotation of d-W8–K9 pair, the side chain of d-W8 is tilted with the main chain and inserts into a different hydrophobic pocket formed by M119^3.29^, T194^ECL2^, I195^ECL2^ and F208^5.38^, thus losing contact with helix VI that dominates the activation of class A GPCRs (Fig. 3c). Besides the d-W8–K9 pair, other residues of CYN 154806 also form different contacts with the receptor. Unlike the stretched conformation of F7 of SST-14 in SSTR2, Y7 of CYN 154806 is flipped to the opposite side to form hydrophobic interactions with the d-Y12 of CYN 154806, constraining the β turn conformation of the peptide (Fig. 3b, c). This conformational change might be caused by the chirality difference between l- and d-residues at the fifth and sixth residues of CYN 154806 and results in a different binding mode compared to that of SST-14. The flipped Y7 of CYN 154806 forms minor hydrophobic interactions with T194^ECL2^ while the side chain of d-Y12 makes new hydrophobic interactions with Q102^2.63^, V103^2.64^ and E106^ECL1^ (Fig. 3c, d). The first residue of CYN 154806, NO_2_-F5, is accommodated by a similar environment to F6 of SST-14 in SSTR2, however, its C-terminus amidated residue, Y12, forms additional polar interactions with S42^1.31^, N43^1.32^ and D295^7.36^, which are not observed in agonist-bound SSTR2 structures (Fig. 3d). Alanine replacement of these polar residues attenuated the binding of CYN 154806 by ~2 fold (Fig. 3e; Supplementary information, Table S4), demonstrating that these interactions also have a minor role in ligand recognition.

### Non-peptidic agonist binding to SSTR2 and SSTR4

**Fig. 4:**
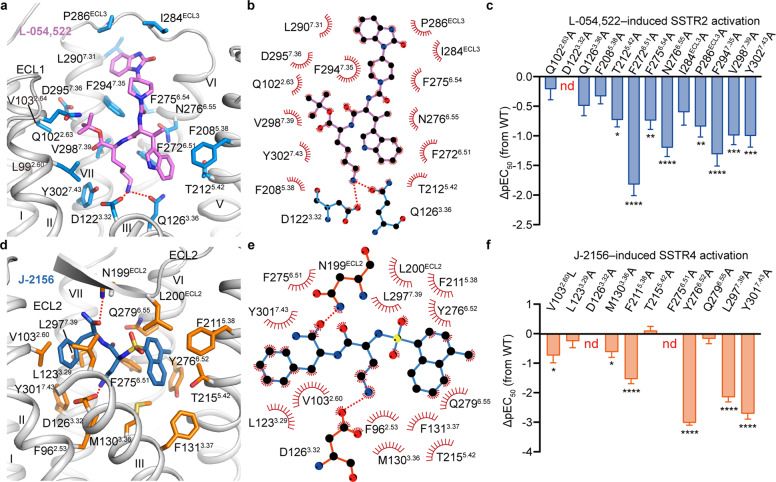
Binding modes of non-peptidic agonists. **a** Interactions between L-054,522 and SSTR2. Involved residues of SSTR2 are shown as sticks and colored by protactinium. L-054,522 is shown as sticks and colored by violet. **b** Schematic representation of the interactions between SSTR2 and L-054,522 analyzed using LigPlot^+^ program.^59^ Atoms are shown as circle and colored by black (carbon), red (oxygen) and blue (nitrogen). Polar interactions are indicated by red dash lines. **c** Inhibition of forskolin-stimulated cAMP accumulation of WT SSTR2 and SSTR2 mutants induced by L-054,522. Bars represent the differences between the calculated L-054,522 potency (pEC_50_) of WT and mutants. Data are shown as means ± SEM from at least three independent experiments. **d** Interactions between J-2156 and SSTR4. Involved residues of SSTR4 are shown as sticks and colored by orange. J-2156 is shown as sticks and colored by sky blue. **e** Schematic representation of the interactions between SSTR4 and J-2156 analyzed using LigPlot^+^ program. **f** Inhibition of forskolin-stimulated cAMP accumulation of WT SSTR4 and SSTR4 mutants induced by J-2156. The *P* value was defined as: *P < 0.05; **P < 0.01; ***P < 0.001; ****P < 0.0001. nd (not determined) indicates that a robust concentration response curve could not be determined within the concentration range tested. Detailed statistical evaluation is shown in Supplementary information, Table S3.

L-054,522, which reserves the structural feature of peptide ligands, occupies a binding pocket similar to SST-14 in SSTR2 (Fig. 4a). It slants from helix III to the hydrophobic cavity formed by helices VI, VII and ECL3 of SSTR2 and makes extensive interactions with the receptor (Fig. 4a, b). Anchored by the conserved polar interactions with D122^3.32^ and Q126^3.36^, the chemical group of β-methyl-(d)-W–K of L-054,522 is also involved in similar hydrophobic interactions with conserved residues of SSTR2, including F208^5.38^, T212^5.42^, F272^6.51^, N276^6.55^, V298^7.39^ and Y302^7.43^ (Fig. 4b, c). Structural analysis reveals that an additional β-methyl could improve the affinity of the ligand by enhancing hydrophobic interactions with the receptor, consistent with previous structure-activity relationship (SAR) studies.^16^ The tert-butyl ester of L-054,522 stretches to the hydrophobic pocket formed by helices II and VII, which would strengthen the hydrophobic interactions with L99^2.60^, Q102^2.63^ and V103^2.64^ of SSTR2 (Fig. 4a). SAR studies showed that substitution of methyl with tert-butyl ester could further improve the binding of L-054,522 to SSTRs.^17^ Similarly, 2-oxo-3H-benzimidazole of L-054,522 extends toward the extracellular part and lies down in the hydrophobic cavity formed by helix VI, ECL3 and helix VII (F275^6.54^, I284^ECL3^, P286^ECL3^, L290^7.31^ and F294^7.35^), which is a major determinant for ligand selectivity of SSTR2 (Fig. 4a).

J-2156, a sulfonamide compound without the typical “W–K” pair, adopts a totally different binding pose in SSTR4 compared to that of L-054,522 in SSTR2, with a β-methyl-naphthyl which mimics the side chain of W8 extending to helix V and a phenyl ring on the opposite side approaching to a sub-pocket formed by helices II and III that is absent in SSTR2 (Fig. 4d). The protonated amino group covalently linked by ethyl group forms a salt bridge with D126^3.32^, which mimics the side chain of K9 of SST-14 (Fig. 4d, e). Owing to the rigid chemical skeleton of J-2156, β-methyl-naphthyl is compacted tightly with M130^3.36^, F131^3.37^, T215^5.42^, F275^6.51^, Y276^6.52^ and Q279^6.55^ (Fig. 4d). Alanine mutations of these residues compromised the EC_50_ of J-2156, especially for F275^6.51^ and Y276^6.52^ which form extensive hydrophobic packing with β-methyl-naphthyl, and cause a significant reduction in agonist potency, indicating an important role of these residues in ligand-induced receptor activation (Fig. 4d–f; Supplementary information, Table S3). The phenyl ring of J-2156 is sitting in the sub-pocket formed by helices II and III, including V103^2.60^, L123^3.29^ and L297^7.39^ (Fig. 4d). Replacement of the phenyl ring with diphenyl methyl caused a steric collision in this sub-pocket and compromised the ligand affinity by ~4-fold.^33^ Sequence alignment reveals a remarkable difference in positions 2.60 and 3.29 among different SSTRs (Supplementary information, Fig. S6), which might be a key factor governing the selectivity of J-2156 for SSTR4. V103^2.60^ and L123^3.29^ are present as leucine and methionine, respectively, in other SSTRs. The larger side chains of leucine and methionine might cause spatial hindrance and be sterically incompatible with the phenyl ring of J-2156. Indeed, mutating V103^2.60^ to leucine decreased the potency of J-2156 by ~5-fold (Fig. 4f; Supplementary information, Table S3).

### Activation mechanism of SSTRs

To reveal the activation mechanism of SSTRs, the structures of SST-14–SSTR2–G_i1_, SSTR2–L-054,522 and SSTR2–CYN 154806 complexes are superimposed. Compared with SSTR2–CYN 154806, the extracellular part of agonist-bound SSTR2 is more compact, which has been observed in the fully active structure of μ opioid receptor (μ-OR) in comparison with its antagonist-bound states (Supplementary information, Fig. S7a).^34^ Unlike CYN 154806, the agonists are shifting more toward helix III, leading to the retraction of Q126^3.36^ (Supplementary information, Fig. S7b). This coincides with the indole ring of W269^6.48^ directly and turns on the “toggle-switch” that is essential for GPCR activation (Supplementary information, Fig. S7b). W269^6.48^ induces a rearrangement of the “P^5.50^–I^3.40^–F^6.44^” motif of SSTR2 and initiates the outward movement of helix VI and inward movement of helix V (Supplementary information, Fig. S7c). Additionally, a striking side chain rotation of N125^3.35^ occurs in agonist-bound structures disrupting the hydrogen bond network formed by D89^2.50^, N125^3.35^ and S305^7.46^. This leads to the collapse of the interaction between helices III and VII and arouses the rearrangement of the “NPxxY” motif (Supplementary information, Fig. S7d). To allow insertion of α5 helix of Gα_i_, the intrahelical ionic lock between D139^3.49^ and R140^3.50^ is broken and R140^3.50^ is released to form the hydrogen network with Y228^5.58^ and Y312^7.53^ to stabilize the cytoplasmic conformation for G protein coupling (Supplementary information, Fig. S7e). This phenomenon was also observed in SSTR4–G_i1_ complexes when compared to SSTR2–CYN 154806 (Supplementary information, Fig. S7f–i), suggesting a conserved activation mechanism of SSTRs.

Compared to the SST-14–SSTR2 structure, binding of CYN 154806 induces a much larger pocket in SSTR2. A 70-degree counterclockwise rotation leads to a higher position of d-W8–K9 pair, with side chain of W8 pushing the ECL2 outward (Fig. 3b; Supplementary information, Fig. S7a). F5 side chain of CYN 154806 inserts into a highly conserved hydrophobic cavity, and its NO_2_ modification induces an outward movement of helix VI, ECL3 and helix VII (Fig. 3d; Supplementary information, Fig. S7a). It is known that inverting the residues chirality of d-F5 and C6 of octreotide could convert an agonist to an antagonist.^14,32^ The chirality inversion of C6 to d-C6 in CYN 154806 induces a different packing of Y7 and Y12, which pushes helix II outward (Fig. 3c, d; Supplementary information, Fig. S7a). It is widely accepted that activation of GPCRs involves a contraction of the helical bundle. The chirality inversion in CYN 154806 resulted in a concomitant outward movement of extracellular tips of helices II, VI and VII compared to that of agonist-bound SSTR2, and this might antagonize SSTR2 by expanding the ligand-binding pocket (Supplementary information, Fig. S7a). In addition, the salt bridge between D122^3.32^ and K9 pulls the helix III upward and creates a kink, further preventing receptor activation through inhibiting the conformational change of Q126^3.36^ (Supplementary information, Fig. S7b).

### Subtype selectivity of SSTR family

Due to their diverse pharmacological properties and expression patterns, highly selective SSTR ligands are critical for drug development. A large variety of SST analogs and small-molecule ligands have thus been made with different specificities for SSTRs. Octreotide (d-F5–C6–F7–d-W8–K9–T10–C11–T12-ol, disulfide bridge: C6–C11, numbered as in SST-14) with a six-residue ring structure showed good selectivity for SSTR2 but also binds to SSTR3 and SSTR5.^35^ In contrast, peptide 3 (C5–F6–A7–W8–K9–T10–F11–C12-OH, disulfide bridge: C5–C12) was identified as a SSTR4-selective agonist with two additional residues between the disulfide bond (Fig. 3a). Alanine substitution at position 7 of peptide 3 was also introduced to improve ligand selectivity for SSTR4 over SSTR2.^36^ To unveil the details of octreotide selectivity for different SSTR subtypes, molecular docking and dynamics simulation studies were carried out based on the structures of SSTR2 and SSTR4.

**Fig. 5:**
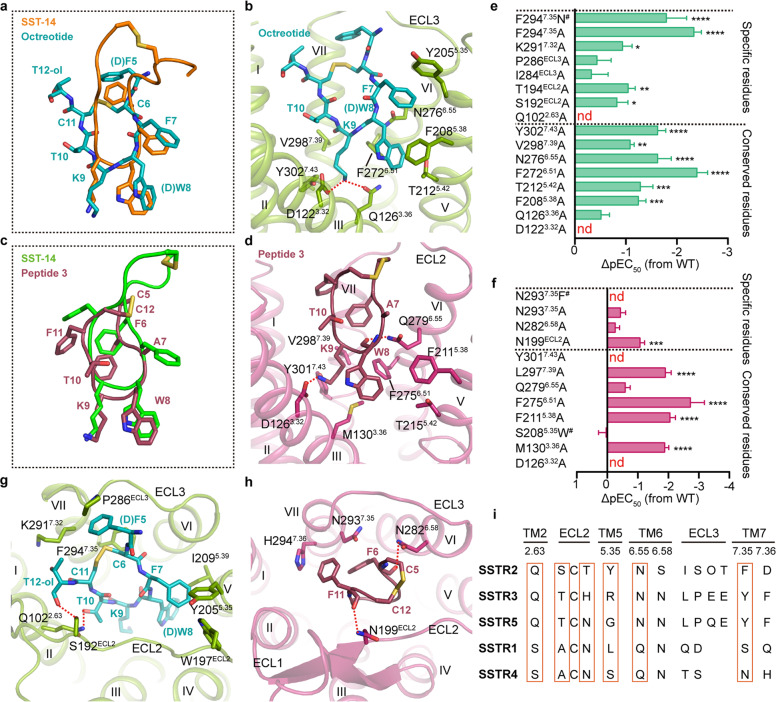
Subtype selectivity between SSTR2 and SSTR4. **a** Comparison of the binding mode of octreotide in the docking model and that of SST-14 in the SST-14–SSTR2 complex. Octreotide is shown as teal sticks and SST-14 is shown as orange cartoon and sticks. **b** Interactions between the key pharmacophore (F7–d-W8–K9–T10) of octreotide and SSTR2. SSTR2 is shown as split-pea cartoon and involved residues are shown as sticks. Polar interactions are indicated by red dash lines. **c** Comparison of the binding mode of peptide 3 in simulation snapshots of the peptide 3–SSTR4 complex and that of SST-14 in the SST-14–SSTR4 complex. Peptide 3 is shown as raspberry sticks and SST-14 is shown as green cartoon. **d** Interactions between the key pharmacophore (A7–W8–K9–T10) of peptide 3 and SSTR4. SSTR4 is shown as warm-pink cartoon and involved residues are shown as sticks. **e**, **f** Inhibition of forskolin-stimulated cAMP accumulation of WT SSTR2 and SSTR2 mutants induced by octreotide (**e**) or that of WT SSTR4 and SSTR4 mutants induced by peptide 3 (**f**). Bars represent the differences between the calculated agonist potency (pEC_50_) of WT and mutants. The *P* value was defined as: *P < 0.05; **P < 0.01; ***P < 0.001; ****P < 0.0001. nd (not determined) indicates that a robust concentration response curve could not be determined within the concentration range tested. ^#^Low surface expression level (< 40% of WT expression). Detailed statistical evaluation is shown in Supplementary information, Table S3. **g**, **h** Interactions between octreotide and SSTR2 (**g**) or between peptide 3 and SSTR4 (**h**). **i** Sequence alignment of SSTRs. Orange boxes indicate specific residues involved in selective ligand binding.

In the octreotide–SSTR2 model, octreotide shares the similar binding pose with SST-14 in SSTR2. The pharmacophore (F7–d-W8–K9–T10) of octreotide was shown to bind at the bottom of the binding pocket as seen in SST-14–SSTR2 but with a slight conformational difference in the residues outside the pharmacophore (Fig. 5a, b). Mutagenesis studies verified that alanine mutation of D122^3.32^, Q126^3.36^, F208^5.38^, T212^5.42^ and N276^6.55^, V298^7.39^ and Y301^7.43^ reduced the EC_50_ values of octreotide (Fig. 5e; Supplementary information, Table S3). For peptide 3, the simulation data reveal that its overall RMSD is much smaller in SSTR4 than that in SSTR2, indicating that peptide 3 is structurally stable upon binding to SSTR4. In addition, both of SSTR2 and SSTR4 themselves are also structurally stable with an RMSD of Cα of TMD within 4 Å (Supplementary information, Fig. S8a, b). After 1000-ns simulation, peptide 3 still adopted a similar conformation as SST-14 in SSTR4 with the pharmacophore (A7–W8–K9–T10) forming conserved interactions with SSTR4 (Fig. 5c, d). The side chains of F6 and F11 of peptide 3 in SSTR4 packed together to stabilize the conformation of each other while the hydrophobic interactions between F6 and F11 of peptide 3 in SSTR2 were broken (Fig. 5d; Supplementary information, Fig. S8c). To confirm these simulation results, the conserved interactions between peptide 3 and SSTR4 were mutated and impaired peptide 3-induced activity was observed as predicted by our simulation results (Fig. 5f; Supplementary information, Table S3).

According to the docking and simulation results, d-F5 and disulfide bond of the octreotide protrude into the hydrophobic cavity formed by helix VI, ECL3 and helix VII, which is specific for SSTR2, contributing to its restrictive and smaller ring structure (Fig. 5g). Alanine replacement of d-F5 eliminated the hydrophobic interactions with this cavity formed by P286^ECL3^, K291^7.32^ and F294^7.35^ and strikingly decreased the binding of octreotide to SSTR2.^37^ In addition, as observed in SST-14-bound SSTR4, the shorter ECL3 and helix VII and the polar surrounding of this region in SRIF2 form spatial hindrance to octreotide and lead to diminished affinities of SST analogs sharing a similar architecture with octreotide to these two receptors (Supplementary information, Fig. S6). F6 and F11 of peptide 3 are surrounded by the side chains of N293^7.35^ and H294^7.36^ (Fig. 5h). N293^7.35^F totally abolished the activity of peptide 3, indicating that spatial clash is not tolerable at this position (Fig. 5f; Supplementary information, Table S3). Besides peptide analogs, superimposition of L-054,522 in SSTR4 also showed severe spatial clashes between 2-oxo-3H-benzimidazole of L-054,522 and the shorter ECL3 and helix VII of SSTR4, blocking the binding of L-054,522 to SSTR4 (Supplementary information, Fig. S6). Taken together, these observations suggest that the hydrophobic cavity formed by helix VI, ECL3 and helix VII in SSTR2 could be one of the major target sites for developing SRIF1-selective ligands.

In SSTR4, the phenyl ring of J-2156 sits in the hydrophobic sub-pocket formed by helices II, III and ECL1 (Supplementary information, Fig. S9a). This sub-pocket is also observed in SST-14-bound SSTR4, although not occupied by the peptide (Supplementary information, Fig. S9b). However, similar sub-pocket at the corresponding position was not found in any of the SSTR2 structures (Supplementary information, Fig. S9c, d). Even though a relatively small cavity was observed in SST-14–SSTR2 complex structure, it is too small to serve as a sub-pocket to contribute to ligand selectivity in comparison with that in SSTR4 (~40 Å^3^ vs ~80 Å^3^ as calculated by CASTp in Chimera). Bulker residues leucine and methionine are present in positions 2.60 and 3.29 of SSTR2 and other SRIF1 subfamily receptors (Supplementary information, Fig. S6), which form hydrophobic interactions with each other and prevent the sub-pocket from accommodating the phenyl ring of J-2156 (Supplementary information, Fig. S9c, d). Therefore, the difference in this sub-pocket between different SSTR subtypes explains the selectivity of J-2156. Thus, increasing the flexibility by enlarging the ring size of the cyclic peptides enables the ligands to accommodate the ligand-binding pocket of SSTR4. Ligand selectivity could also be improved by introducing modifications targeting this sub-pocket.

Structural analysis demonstrated divergent upper portions of the receptors which might contribute to subtype selectivity as well. Two hydrogen bonds were observed between Q102^2.63^, S192^ECL2^ of SSTR2 and the N-terminus and T10 of octreotide. However, they are not conserved in other SSTRs (Fig. 5g, i). Previous studies showed that swapping the ECL2 of SSTR2 with that of SSTR1 decreased octreotide binding by ~40-fold. Consistent with our prediction, alanine mutation of Q102^2.63^ and S192^ECL2^ reduced octreotide-induced bioactivity. Peptide 3 has additional two hydrogen bonds with N199^ECL2^ and Q279^6.55^ in SSTR4; however, no interaction was observed between peptide 3 and the corresponding residues in SSTR2 during the simulation as they were replaced by threonine and asparagine (Fig. 5d, h; Supplementary information, Fig. S8d, e). Such interactions may also contribute to peptide selectivity for SSTR4 over SSTR2. Y205^5.35^ of SSTR2 is present as serine in SSTR4, which could be another contributor to ligand selectivity (Fig. 5i). A previous study showed that phenylalanine substitution at position 7 of peptide 3 could enhance the ligand binding to SSTR2 by ~6-fold but had little effect on SSTR4.^36^ Based on our simulation results, phenylalanine substitution at position 7 of peptide 3 might introduce extra hydrophobic interactions with Y205^5.35^ of SSTR2, while neither alanine nor phenylalanine showed obvious interaction with S208^5.35^ of SSTR4 (Supplementary information, Fig. S8f). This might explain the fact that alanine substitution at position 7 of peptide 3 had a higher selectivity for SSTR4 with reduced binding ability to SSTR2.

## Discussion

SSTRs are invaluable drug targets for the pharmacological treatment of NETs and abnormal hormone secretion. Investigating the molecular basis underlying ligand selectivity of each SSTR subtype would facilitate the structure-based design of drugs with better efficacy against SSTRs. In this study, we present five structures of SSTR2 and SSTR4 bound to different types of selective ligands with different states. Combined with mutagenesis, molecular docking and simulation studies, all of the studied ligands are shown to occupy similar environment and form conserved interactions at the bottom of the binding pocket, revealing a molecular basis of the ligand–receptor recognition (Supplementary information, Fig. S10). Notably, sequence alignment revealed divergent upper portions of the ligand-binding pockets among SSTRs which might lead to ligand selectivity (Supplementary information, Fig. S10a, c). The hydrophobic cavity formed by helix VI, ECL3 and helix VII as well as the non-conserved residues of helix II and ECL2 contribute to the ligand selectivity for SSTR2 (Supplementary information, Fig. S10a, b). On the other hand, small-molecule agonist J-2156-bound SSTR4 presents a sub-pocket formed by helices II and III which is disrupted in SSTR2, revealing a possibility to design selective ligand targeting this sub-pocket (Supplementary information, Fig. S10c, d). The antagonist CYN 154806- and agonist L-054,522-bound SSTR2 structures were determined in artificial membrane bilayers, while cryo-EM structures of SST-14-bound SSTR2 and SSTR4 were solved in fully active states with detergents. We cannot exclude the possibility that different lipid environments might contribute to conformational alterations in comparison with structures obtained by different methods. However, the different conformational states observed in this study are unlikely due to this reason, given that the published structures of agonist-bound GPCR complexes (e.g., FPR2) solved by X-ray crystallography (in lipid environment) and cryo-EM (in detergent environment) represent a similar active conformation of the receptors.^38,39^ Structural comparison reveals the activation mechanism of the receptors as well as antagonism of the ligands. Upon agonist binding, conformational change of Q126^3.36^ initiates the receptor activation by coinciding with W228^6.48^, while CYN 154806 prevents this key conformational change to exert antagonism.

In general, our work provides molecular mechanisms of ligand recognition, subtype selectivity, receptor activation and G protein coupling among SSTRs from the perspective of structural biology, thereby offering new opportunities to design better therapeutics against this important family of drug targets.

## Materials and methods

### Construct cloning and expression

For crystallization, the WT human gene *SSTR2* was cloned into a modified pFastBac1 vector (Invitrogen) containing an expression cassette with a haemagglutinin (HA) signal peptide followed by a Flag tag at the N-terminus and a PreScission protease site followed by a 10× His tag at the C-terminus. The flexible C-terminus was truncated after L360. Three mutations (D89^2.50^N, V106^ECL1^E and S316^7.57^D) were introduced and residues 238–243 were removed and replaced by xylanase to crystallize with CYN 154806, while D89^2.50^N was reinstated and the junction site was optimized between 240 and 242 to crystallize with L-054,522. High-titer recombinant viruses (> 10^8^ viral particles/mL) were obtained using the Bac-to-Bac baculovirus expression system (Invitrogen). *Spodoptera frugiperda* (*Sf*9) cells at density of 2 × 10^6^ to 3 × 10^6^ cells/mL were infected with the virus at multiplicity of infection (MOI) of 5. Cells were collected by centrifugation at 48 h postinfection and stored at –80 °C until use.

For cryo-EM, the WT human genes *SSTR2* and *SSTR4* were cloned into a modified pFastBac1 vector with an HA signal peptide followed by a Flag tag at the N-terminus and a PreScission protease site followed by a twin-strep tag at the C-terminus. To facilitate stable complex formation, the C-termini of SSTR2 and SSTR4 were truncated after L359 and L328, respectively. One mutation, V^6.40^F, was introduced to help the SSTR4−G_i1_ complex formation. The dominant-negative *Gα*_*i1*_ (*DNG*_*i1*_) with five mutations (S47C, G202T, G203A, E245A and A326S) was cloned into the modified pFastbac1 vector.^40^ The human genes *Gβ*_*1*_ and *Gγ*_*2*_ were cloned into the pFastBac Dual vector (Invitrogen) with a 6× His tag at the N-terminus of *Gβ*_*1*_. The gene *scFv16* was cloned into the modified pFastBac1 vector with an N-terminal GP67 signal peptide and a C-terminal 8× His tag. High-titer recombinant viruses were obtained using the Bac-to-Bac baculovirus expression system. High Five insect cells at a density of 1.5 × 10^6^ cells/mL were transfected with modified *SSTR2* or *SSTR4, Gα*_*i1*_ and *Gβ*_*1*_*γ*_*2*_ at the MOI ratio of 1:1:1 and cells were collected by centrifugation after 48 h postinfection and stored at –80 °C until use. *ScFv16* was expressed using High Five insect cells and the culture supernatant was collected after 48 h post infection.

### Purification of SSTR2 for crystallization

Insect cell membranes were disrupted by thawing frozen cell pellets in a hypotonic buffer containing 10 mM HEPES, pH 7.5, 10 mM MgCl_2_, 20 mM KCl and EDTA-free complete protease inhibitor cocktail (Roche) followed by repeated dounce homogenization. Extensive washing of the membranes was performed by centrifugation in the same hypotonic buffer, followed by a high osmotic buffer containing 1 M NaCl, 10 mM HEPES, pH 7.5, 10 mM MgCl_2_, 20 mM KCl (three times), and then the hypotonic buffer to remove high concentration of NaCl. Purified membranes were resuspended in 10 mM HEPES, pH 7.5, 30% (v/v) glycerol, 10 mM MgCl_2_, 20 mM KCl and EDTA-free complete protease inhibitor cocktail, flash frozen in liquid nitrogen and stored at –80 °C until use.

Purified membranes were thawed on ice in the presence of 1 mg/mL CYN 154806 or 100 μM L-054,522, 2 mg/mL iodoacetamide, EDTA-free protease inhibitor cocktail (Roche), and incubated at 4 °C for 30 min before solubilization. SSTR2 protein was extracted from the membrane by adding *n*-dodecyl-β-d-maltopyranoside (DDM; Affymetrix) and cholesteryl hemisuccinate (CHS; Sigma) to the membrane solution to a final concentration of 0.5% (w/v) and 0.1% (w/v), respectively, followed by continued stirring at 4 °C for 3 h. The supernatant was isolated by centrifugation at 160,000× *g* for 30 min and incubated with TALON IMAC resin (Clontech) overnight at 4 °C. The resin was then washed with twenty column volumes of 50 mM HEPES, pH 7.5, 0.8 M NaCl, 10% (v/v) glycerol, 0.05% (w/v) DDM, 0.01% (w/v) CHS and 30 mM imidazole in the presence of 1 mg/mL CYN 154806 or 100 μM L-054,522, and eluted with 5 column volumes of 50 mM HEPES, pH 7.5, 0.8 M NaCl, 10% (v/v) glycerol, 0.05% (w/v) DDM, 0.01% (w/v) CHS and 300 mM imidazole in the presence of 1 mg/mL CYN 154806 or 100 μM L-054,522. PD MiniTrap G-25 column (GE Healthcare) was used to remove imidazole. The protein was further purified by the treatment with His-tagged PreScission protease and PNGase F and then concentrated to 20–30 mg/mL with a 100 kDa molecular weight cut-off concentrator (Millipore).

### Crystallization of SSTR2–CYN 154806 and SSTR2–L-054,522 complexes

Purified SSTR2 samples were reconstituted in LCP by mixing with molten lipid in a mechanical syringe mixer. The protein solution was mixed with monoolein/cholesterol (10:1 by mass) lipids at a weight ratio of 1:1.5 (protein:lipid). After formation of a transparent LCP, the mixture was dispensed onto 96-well glass sandwich plates (Shanghai FAstal BioTech) in 40–50 nL drops and overlaid with 800 nL precipitant solution using a Mosquito LCP robot (TTP Labtech). Protein reconstitution in LCP and crystallization trials were performed at room temperature (RT, 19–22 °C). Plates were incubated and imaged at 20 °C using an automated incubator/imager (RockImager, Formulatrix). The SSTR2−CYN 154806 complex was crystallized in 100–300 mM ammonium sulfate, 6%–10% PEG2000, 1 mg/mL CYN 154806, and 0.1 M HEPES, pH 7.0, and the crystals reached their maximum size (100–120 μm) within 2 weeks. The SSTR2−L-054,522 complex was crystallized in 100–400 mM lithium nitrate, 6%–10% PEG2000, 100 μM L-054,522, and 0.1 M HEPES, pH 7.0, and the crystals reached their maximum size (30 μm) within two weeks. The SSTR2 crystals were collected directly from LCP using 50–100 mm micromounts (M2-L19-50/100, MiTeGen) and flash frozen in liquid nitrogen.

### X-ray data collection and structure determination

X-ray diffraction data were collected at the SPring-8 beam line 41XU, Hyogo, Japan, using a Pilatus3 6 M detector (X-ray wavelength 1.0000 Å). The crystals were exposed to a 10-mm mini-beam for 0.5 s and 0.5° oscillation per frame. XDS was used for integrating and scaling data from 11 crystals of the SSTR2–CYN 154806 complex and 24 crystals of the SSTR2–L-054,522 complex.^41^ Initial phase information of the SSTR2−CYN 154806 complex was obtained by molecular replacement (MR) with Phaser using the receptor portion of δ-OR (PDB ID: 4N6H) and xylanase structures (PDB ID: 2B45) as search models.^42^ Subsequently, the model was rebuilt and refined using COOT and PHENIX, respectively.^43,44^ The SSTR2–L-054,522 structure was determined by MR, using the SSTR2–CYN 154806 structure, and subsequently rebuilt and refined as described above.

### Purification of scFv16 and SSTR2–G_i1_/SSTR4–G_i1_ complexes

ScFv16 was balanced with 20 mM Tris-HCl, pH 8.0, 5 mM CaCl_2_ and 1 mM NiCl_2_ at 4 °C for 1 h. Supernatant was then collected and incubated with TALON IMAC resin (Clontech) overnight at 4 °C. The resin was washed with 30 column volumes of 1× PBS, pH 7.4, 10 mM imidazole and eluted with 5 column volumes of 1× PBS, pH 7.4, 300 mM imidazole. The scFv16 was concentrated to 500 μL with a 10 kDa molecular weight cut-off concentrator and further purified by size-exclusive chromatography using a Superdex 75 10/300 GL column (GE Healthcare) to collect monomeric fractions and concentrate to 10 mg/mL for future use.

Insect cell membranes were homogenized in a lysis buffer containing 25 mM HEPES, pH 7.5, 150 mM NaCl, 10 mM MgCl_2_, 10% (v/v) glycerol and EDTA-free complete protease inhibitor cocktail. To assemble the complex in the membranes, 50 μM SST-14 or J-2156, 50 μM TCEP, 100 mU/mL apyrase (NEB) were added to the membranes and stirred at 20 °C for 1 h. Additional 50 μg/mL scFv16 was added to the SSTR4–G_i1_ complexes. The complex proteins were then extracted from the membrane by adding 0.5% (w/v) lauryl maltoseneopentyl glycol (LMNG, Anatrace), 0.05% (w/v) CHS at 4 °C for 2 h. The supernatant was collected by centrifugation at 160,000× *g* for 30 min and incubated with Strep-Tactin XT Superflow resin (IBS lifesciences) at 4 °C overnight. The resin was washed with 20 column volumes of 25 mM HEPES, pH 7.5, 150 mM NaCl, 2 mM MgCl_2_, 0.01% (w/v) LMNG, 0.002% (w/v) CHS and 25 μM SST-14 or J-2156. Then the complexes were eluted with 5 column volumes of 25 mM HEPES, pH 7.5, 150 mM NaCl, 2 mM MgCl_2_, 0.01% (w/v) LMNG, 0.002% (w/v) CHS and 50 μM SST-14 or J-2156. Purified complexes were concentrated thereafter to 500 μL with a 100 kDa molecular weight cut-off concentrator and further purified by size-exclusive chromatography using a Superdex 200 10/300 GL column (GE Healthcare) to collect monomeric fractions and concentrate to 3 mg/mL for data collection.

### Cryo-EM data collection

The purified SST-14–SSTR2–G_i1_, SST-14–SSTR4–G_i1_ and J-2156–SSTR4–G_i1_ complexes were diluted to 1.0, 1.0 and 1.5 mg/mL, respectively. Samples were applied to glow-discharged holey carbon grids (CryoMatrix M024-Au300-R12/13) and then blotted at 4 °C, 100% humidity with force of 0 and blot time of 1.0, 0.5 and 0.5 s, respectively. The grids were plunge-frozen in liquid ethane using the Vitrobot Mark IV (Thermo Fisher Scientific) and stored in liquid nitrogen. Images were obtained by a Titan Krios G3 electron microscope (FEI) of 300 kV with K3 Summit direct electron detector (Gatan) using pixel size of 1.045. A total of 9187, 9947 and 5427 movie stacks were recorded with defocus ranging from –1.3 to –2.3 μm, exposing to a dose rate of 1.875 electrons/Å^2^/frame. Each movie stack contains 40 frames for a total dose of 70 electrons/Å^2^. SerialEM was applied to automated single-particle data acquisition.^45^

### Cryo-EM data processing

Collected movies were subjected to beam-induced motion correction and contrast transfer function (CTF) determination by MotionCor2 and Gctf v1.18.^46,47^ A total of 3,185,362, 4,096,528 and 2,338,149 particles for the SST-14–SSTR2–G_i1_, SST-14–SSTR4–G_i1_ and J-2156–SSTR4–G_i1_ complexes were auto-picked guided by RELION3.1 and Gautomatch v0.56 (developed by K. Zhang, MRC Laboratory of Molecular Biology, Cambridge, UK, http://www.mrc-lmb.cam.ac.uk/kzhang/Gautomatch/) and then subjected to two rounds of reference-free 2D classification to discard false positive particles.^48^ An ab initio model generated by RELION3.1 was used as initial reference model for 3D classification. A total of 990,095, 1,230,425 and 853,618 particles were selected for a further round of 3D classification. The best class with 696,255 particles of the SST-14–SSTR2–G_i1_ complex, 799,646 particles for the SST-14–SSTR4–G_i1_complex and 600,908 particles for J-2156–SSTR4–G_i1_ complex were selected and subjected to 3D auto-refinement in RELION3.1, respectively. The final maps were improved by Bayesian polishing, resulting in 3.1 Å, 2.9 Å and 2.8 Å maps based on the gold-standard Fourier Shell Correlation (FSC) using the 0.143 criterion. The local resolution for these maps was generated by ResMap.^49^

### Cryo-EM model building

The receptors of SST-14–SSTR2–G_i1_ and SST-14/J-2156–SSTR4–G_i1_ complexes were modeled using μ-OR (PDB ID: 6DDE) by SWISS-MODEL. Subunits of G_i_ and scFv16 were built using the components of the glucagon–GCGR–G_i1_ complex (PDB ID: 6LML). Models were fitted to corresponding maps using UCSF Chimera.^50^ Subsequently, models were rebuilt and refined using COOT and PHENIX, respectively. The final models were validated by MolProbity.^51^ Structure figures in this paper were prepared by Pymol (https://pymol.org/2/) and UCSF Chimera.

### Molecular docking

The structure of octreotide was downloaded from PubChem (ID: 448601) and prepared with the Ligprep tool of Schrödinger suite. The ionization state of octreotide was predicted by the Epik module with a pH of 7.5. For the preparation of SSTR2, the Protein Preparation Wizard in the Schrödinger suite was used to add the missing side chains and hydrogen atoms, and the ionizable groups in the receptor were optimized by PROPKA module with a pH of 7.5. The structure of the receptor was then refined under OPLS3 forced field based on the heavy atom’s restraint. The docking of octreotide into SSTR was performed with the Ligand Docking by Glide and all serines/tyrosines/threonines/cysteines potentially interacted with the ligand were selected as the rotatable group during gird generation. Top energy minimized docking poses of octreotide were selected according to GildeScore for the further induced fit docking (IFD).^52^ Induced-fit docking was executed at default settings except the extra-precision mode was selected in the “Glide Redocking”. In the “Prime Refinement” of IFD, all residues at the distance of 5 Å from the ligand pose were refined and no other residues were specified for refinement. The docking grid was centered on the centroid of ligand from the top energy minimized docking pose. The docking pose of octreotide was selected from the high-score conformations for further structural analysis.

### Molecular dynamics simulation

Molecular dynamics simulation studies were performed by Gromacs 2020.1.^53^ The receptor was prepared and capped by the Protein Preparation Wizard (Schrodinger 2017-4). Given the high sequence similarity between SST-14 and peptide 3, the model of the peptide 3-bound SSTR2/SSTR4 was built on the basis of the cryo-EM SST-14–SSTR2/SSTR4–G_i_ complex structures by several rounds of single point mutation using the “Residue and Loop Mutation” module in BioLuminate (Schrodinger 2017-4). Two residues (D^2.50^ and D^3.49^) were deprotonated, while other titratable residues were left in their dominant state at pH 7.0. The complexes were embedded in a bilayer composed of 176–178 POPC lipids and solvated with 0.15 M NaCl in explicit TIP3P waters using CHARMM-GUI Membrane Builder.^54^ The CHARMM36-CAMP force filed was adopted for protein, peptides, lipids and salt ions.^55^ The Particle Mesh Ewald (PME) method was used to treat all electrostatic interactions beyond a cutoff of 10 Å and the bonds involving hydrogen atoms were constrained using LINCS algorithm.^56^ The complex system was first relaxed using the steepest descent energy minimization, followed by slow heating of the system to 310 K with restraints. The restraints adopted from the default setting in the CHARM-GUI webserver v3.2.2 were reduced gradually over 20 ns, with a simulation step of 1 fs.^54^ Finally, 1000 ns restraint-free production run was carried out for each simulation, with a time step of 2 fs in the NPT ensemble at 310 K and 1 bar using the v-rescale thermostat and the semi-isotropic Parrinello-Rahman barostat, respectively.^57,58^ The related pictures were prepared by Pymol.

### cAMP accumulation assay

HEK293F cells were transiently transfected with WT or mutant SSTR2/SSTR4 using transfection reagent (PEI MAX 2000, Polysciences) and incubated at 37 °C in 5% CO_2_. Forty-eight hours after transfection, cells were centrifuged and resuspended in stimulation buffer (Hanks’ balanced salt solution (HBSS) supplemented with 5 mM HEPES, 0.5 mM IBMX and 0.1% (w/v) bovine serum albumin (BSA), pH 7.4) to a density of 200,000 cells/mL and added to 384-well white plates (PerkinElmer, 1000 cells/well). cAMP accumulation was measured by a LANCE Ultra cAMP kit (PerkinElmer) according to the manufacturer’s instructions. In brief, transfected cells were incubated for 40 min in stimulation buffer with 2.5 μL forskolin (SSTR2: 2 μM; SSTR4: 4 μM) and gradient concentration of ligand at RT. The reactions were stopped by addition of lysis buffer containing 5 μL Eu-cAMP tracer and 5 μL ULight-anti-cAMP. Plates were then incubated for 60 min at RT and time-resolved FRET signals were measured at 620 nm and 665 nm by an EnVision multilabel plate reader (PerkinElmer). Data were analyzed in GraphPad PRISM 8 and all values were normalized to the WT for each ligand. All outcomes were repeated at least three times.

### Whole cell binding assay

For SSTR2 and SSTR4, CHO-K1 cells were cultured in F12 medium with 10% FBS and seeded at a density of 30,000 cells/well in Isoplate-96 plates (PerkinElmer). Twenty-four hours after transfection with the WT or mutant SSTR2/SSTR4, CHO-K1 cells were washed twice and incubated with blocking buffer (F12 supplemented with 25 mM HEPES and 0.1% (w/v) BSA, pH 7.4) for 2 h at 37 °C. For homogeneous competition binding, radiolabeled ^125^I-(Tyr^11^) SST (PerkinElmer; SSTR2, 60 pM; SSTR4, 40 pM) and unlabeled peptide at seven decreasing concentrations (SST-14, 10 μM to 10 pM; L-0545,22, 10 μM to 85 pM; octreotide, 10 μM to 85 pM; CYN 154806, 10 μM to 85 pM; J-2156, 10 μM to 10 pM; peptide 3, 10 μM to 10 pM) were added and competitively reacted with the cells in blocking buffer at RT for 3 h. Following incubation, cells were washed three times with ice-cold PBS and lysed by 50 μL lysis buffer (PBS supplemented with 20 mM Tris-HCl, 1% Triton X-100, pH 7.4). The radioactivity was subsequently counted (counts/min, CPM) in a scintillation counter (MicroBeta^2^ Plate Counter, PerkinElmer) using a scintillation cocktail (OptiPhaseSuperMix, PerkinElmer). Data were analyzed by nonlinear regression using GraphPad PRISM 8.

## Supplementary information

Supplementary information, Figure S1

Supplementary information, Figure S2

Supplementary information, Figure S3

Supplementary information, Figure S4

Supplementary information, Figure S5

Supplementary information, Figure S6

Supplementary information, Figure S7

Supplementary information, Figure S8

Supplementary information, Figure S9

Supplementary information, Figure S10

Supplementary information, Table S1

Supplementary information, Table S2

Supplementary information, Table S3

Supplementary information, Table S4

## Data Availability

Atomic coordinates for the SSTR2–L-054,522 and SSTR2–CYN 154806 structures have been deposited in the RCSB PDB under accession codes 7XN9 and 7XNA. Atomic coordinates and cryo-EM density maps for the SST-14–SSTR2–G_i1_, SST-14–SSTR4–G_i1_ and J-2156–SSTR4–G_i1_ complex structures have been deposited in the PDB under identification codes 7XMR, 7XMS and 7XMT, respectively, and in the Electron Microscopy Data Bank under accession codes EMD-33302, EMD-33303 and EMD-33304, respectively.
