# Supplementary material for: Structural insights into ligand recognition and selectivity of somatostatin receptors
Source: Cell Res. 2022 Jun 23;32(8):761–72. doi: 10.1038/s41422-022-00679-x (PMC9343605; doi:10.1038/s41422-022-00679-x)
Supplement: Supplementary file 1 — Supplementary information, Figure S1 [file 41422_2022_679_MOESM1_ESM.pdf]

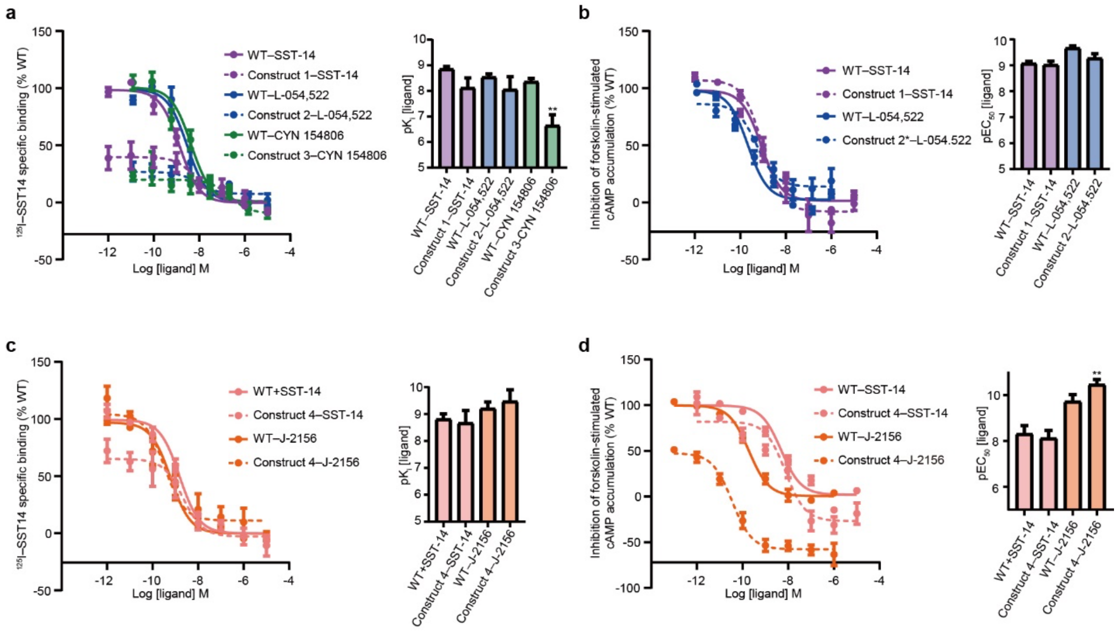

Supplementary information, Fig. S1| Functional validation of structural constructs.

a, Ligand binding of wild-type (WT) and structural constructs of SSTR2 in competition with ^{125}I-SST-14. Construct 1 indicates the modified SSTR2 for cryo-EM structural determination with SST-14. Construct 2 indicates the modified SSTR2 for crystal structural determination with L-054,522. Construct 3 indicates the modified SSTR2 for crystallized structural determination with CYN 154806. (WT–SST-14: $pK_i \pm SEM = 8.86 \pm 0.09$; Construct 1–SST-14: $pK_i \pm SEM = 8.12 \pm 0.39$; WT–L-054,522: $pK_i \pm SEM = 8.53 \pm 0.13$; Construct 2–L-054,522: $pK_i \pm SEM = 8.05 \pm 0.50$; WT–CYN 154806: $pK_i \pm SEM = 8.36 \pm 0.13$; Construct 3–CYN 154806: $pK_i \pm SEM = 6.65 \pm 0.41$). **b**, Agonist induced inhibition of forskolin-stimulated cAMP accumulation of SSTR2. Construct 2* indicates the modified SSTR2 for crystallized structural determination with L-054,522 except that ICL3-xylanase fusion was removed to allow G protein coupling. (WT–SST-14: $pEC_{50} \pm SEM = 9.08 \pm 0.07$; Construct 1–SST-14: $pEC_{50} \pm SEM = 9.02 \pm 0.14$; WT–L-054,522: $pEC_{50} \pm SEM = 9.67 \pm 0.08$; Construct 2*–L-054,522: $pEC_{50} \pm SEM = 9.28 \pm 0.17$). **c**, Ligand binding of wild-type (WT) and structural constructs of SSTR4 in competition with ^{125}I-SST-14. Construct 4 indicates the modified SSTR4 for cryo-EM structural determination with SST-14 and J-2156. (WT–SST-14: $pK_i \pm SEM = 8.81 \pm 0.09$; Construct 4–SST-14: $pK_i \pm SEM = 8.67 \pm$

0.27; WT–J-2156: pK_i ± SEM = 9.21 ± 0.14; Construct 4–J-2156: pK_i ± SEM = 9.47 ± 0.25). **d**, Agonist induced inhibition of forskolin-stimulated cAMP accumulation of SSTR4. (WT–SST-14: pEC_{50} ± SEM = 8.31 ± 0.09; Construct 4–SST-14: pEC_{50} ± SEM = 8.13 ± 0.19; WT–J-2156: pEC_{50} ± SEM = 9.73 ± 0.08; Construct 4–J-2156: pEC_{50} ± SEM = 10.46 ± 0.13). All data are shown as mean ± SEM from at least three independent experiments performed in triplicate. Detailed statistical evaluation is shown in Supplementary information, Table S3, S4.
