# Supplementary material for: Structural insights into ligand recognition and selectivity of somatostatin receptors
Source: Cell Res. 2022 Jun 23;32(8):761–72. doi: 10.1038/s41422-022-00679-x (PMC9343605; doi:10.1038/s41422-022-00679-x)
Supplement: Supplementary file 2 — Supplementary information, Figure S2 [file 41422_2022_679_MOESM2_ESM.pdf]

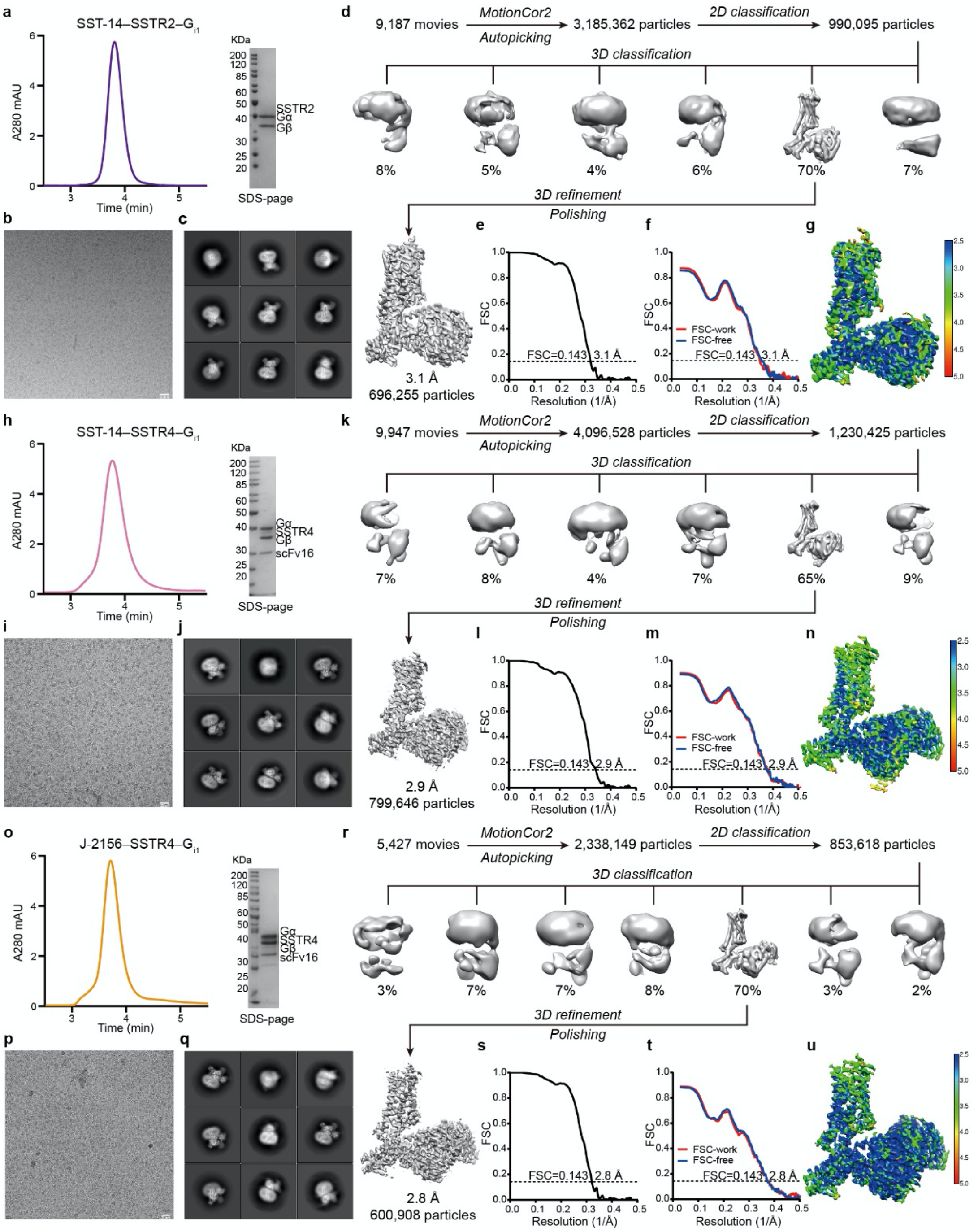

Supplementary information, Fig. S2| Sample preparation and cryo-EM data processing.

a-f, Results of the SST-14–SSTR2–G_{i1} complex. **a**, Analytical size-exclusion chromatography and SDS-PAGE of the purified complex. **b**, **c** Representative cryo-EM images (**b**) and 2D class average image (**c**). **d**, Cryo-EM data procession flow chart of SST-14–SSTR2–G_i complex. **e**, Gold-standard FSC curve showing an overall resolution is 3.1 Å at FSC=0.143. **f**, FSC validation curves for FSC_{work} and FSC_{free} are shown in

red and blue respectively. **g**, Density map according to local resolution estimation. **h-n**, Results of the SST-14–SSTR4–G_i complex. **h**, Analytical size-exclusion chromatography and SDS-PAGE of the purified complex. **i**, **j** Representative cryo-EM image (**i**) and 2D class average image (**j**). **k**, Cryo-EM data procession flow chart of SST-14–SSTR4–G_i complex. **l**, Gold-standard FSC curve showing an overall resolution is 2.9 Å at FSC=0.143. **m**, FSC validation curves for FSC_{work} and FSC_{free} are shown in red and blue respectively. **n**, Density map according to local resolution estimation. **o-u**, Results of the J-2156–SSTR4–G_i complex. **o**, Analytical size-exclusion chromatography and SDS-PAGE of the purified complex. **p, q** Representative cryo-EM image (**p**) and 2D class average image (**q**). **r**, Cryo-EM data procession flow chart of J-2156–SSTR4–G_i complex. **s**, Gold-standard FSC curve showing an overall resolution is 2.8 Å at FSC=0.143. **t**, FSC validation curves for FSC_{work} and FSC_{free} are shown in red and blue respectively. **u**, Density map according to local resolution estimation.
