# Supplementary material for: Structural insights into ligand recognition and selectivity of somatostatin receptors
Source: Cell Res. 2022 Jun 23;32(8):761–72. doi: 10.1038/s41422-022-00679-x (PMC9343605; doi:10.1038/s41422-022-00679-x)
Supplement: Supplementary file 3 — Supplementary information, Figure S3 [file 41422_2022_679_MOESM3_ESM.pdf]

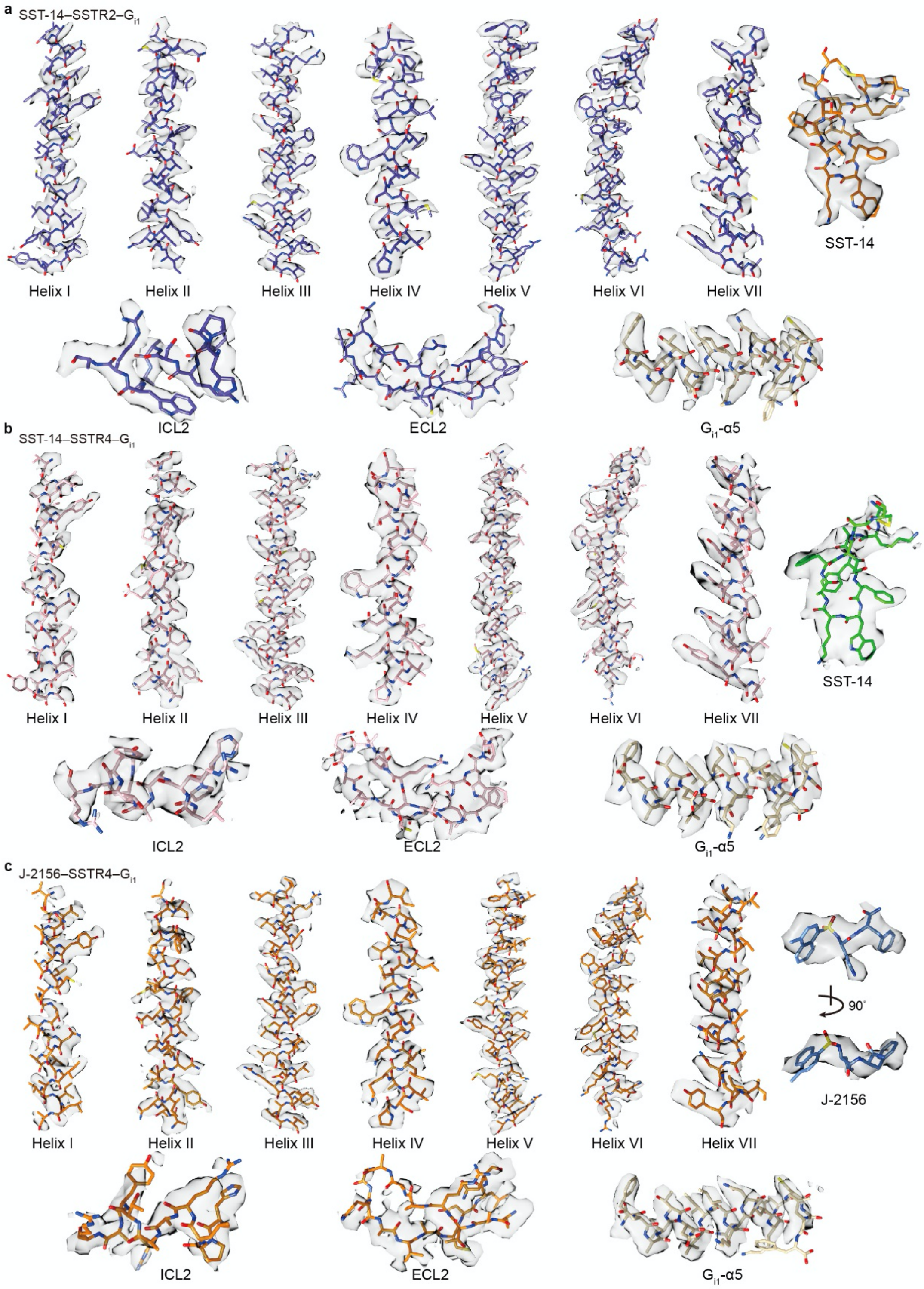

Supplementary information, Fig. S3| Cryo-EM density maps of SSTR2/4–G_i complexes.

a, Cryo-EM density maps and fitted model of all transmembrane helices, ECL2 and ICL2 of SSTR2, SST-14, and α5 helix of $G\alpha_i$. SSTR2 is shown as slate sticks; SST-14

is shown as orange sticks; α5 helix of $G\alpha_i$ is shown as wheat sticks. **b**, Cryo-EM density maps and fitted model of all transmembrane helices, ECL2 and ICL2 of SSTR4, SST-14, and α5 helix of $G\alpha_i$. SSTR4 is shown as pink sticks; SST-14 is shown as green sticks; α5 helix of $G\alpha_i$ is shown as wheat sticks. **c**, Cryo-EM density maps and fitted model of all transmembrane helices, ECL2 and ICL2 of SSTR4, J-2156, and α5 helix of $G\alpha_i$. SSTR4 is shown as orange sticks; J-2156 is shown as sky blue sticks; α5 helix of $G\alpha_i$ is shown as wheat sticks.
