# Supplementary material for: Structural insights into ligand recognition and selectivity of somatostatin receptors
Source: Cell Res. 2022 Jun 23;32(8):761–72. doi: 10.1038/s41422-022-00679-x (PMC9343605; doi:10.1038/s41422-022-00679-x)
Supplement: Supplementary file 4 — Supplementary information, Figure S4 [file 41422_2022_679_MOESM4_ESM.pdf]

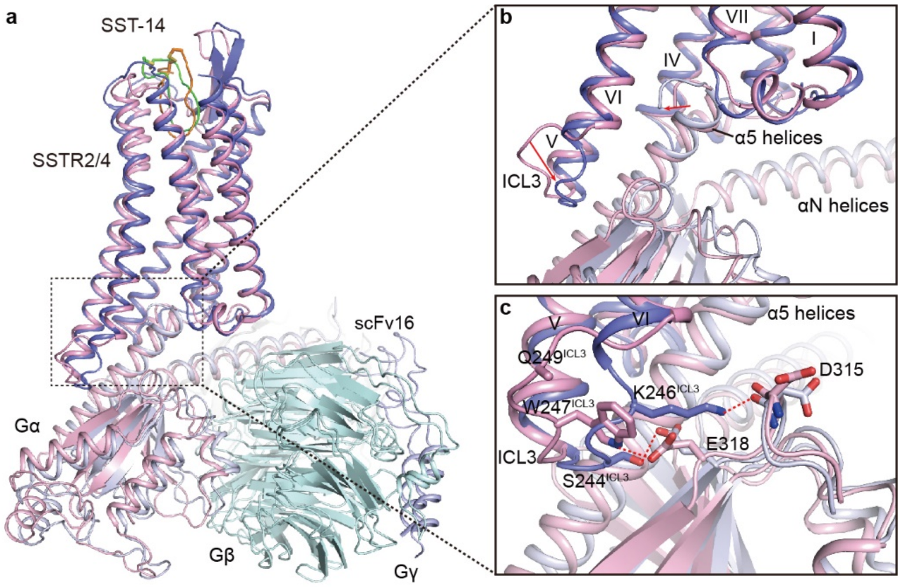

Supplementary information, Fig. S4| G protein coupling of SSTR2–G_i and SSTR4–G_i complexes.

a, Superposition of G_i-bound SSTR2 (SSTR2 in slate cartoon, SSTR2-bound $G\alpha_i$ in blue-white cartoon) and SSTR4 (SSTR4 in pink cartoon, SSTR4-bound $G\alpha_i$ in light-pink cartoon). **b**, Conformational changes of the receptor and α5 helices of $G\alpha_i$ are indicated by red arrows. **c**, Detailed interactions leading to the conformational changes between the receptor and $G\alpha_i$ are shown as sticks. Polar interactions are indicated by red dash lines.
