# Supplementary material for: Structural insights into ligand recognition and selectivity of somatostatin receptors
Source: Cell Res. 2022 Jun 23;32(8):761–72. doi: 10.1038/s41422-022-00679-x (PMC9343605; doi:10.1038/s41422-022-00679-x)
Supplement: Supplementary file 5 — Supplementary information, Figure S5 [file 41422_2022_679_MOESM5_ESM.pdf]

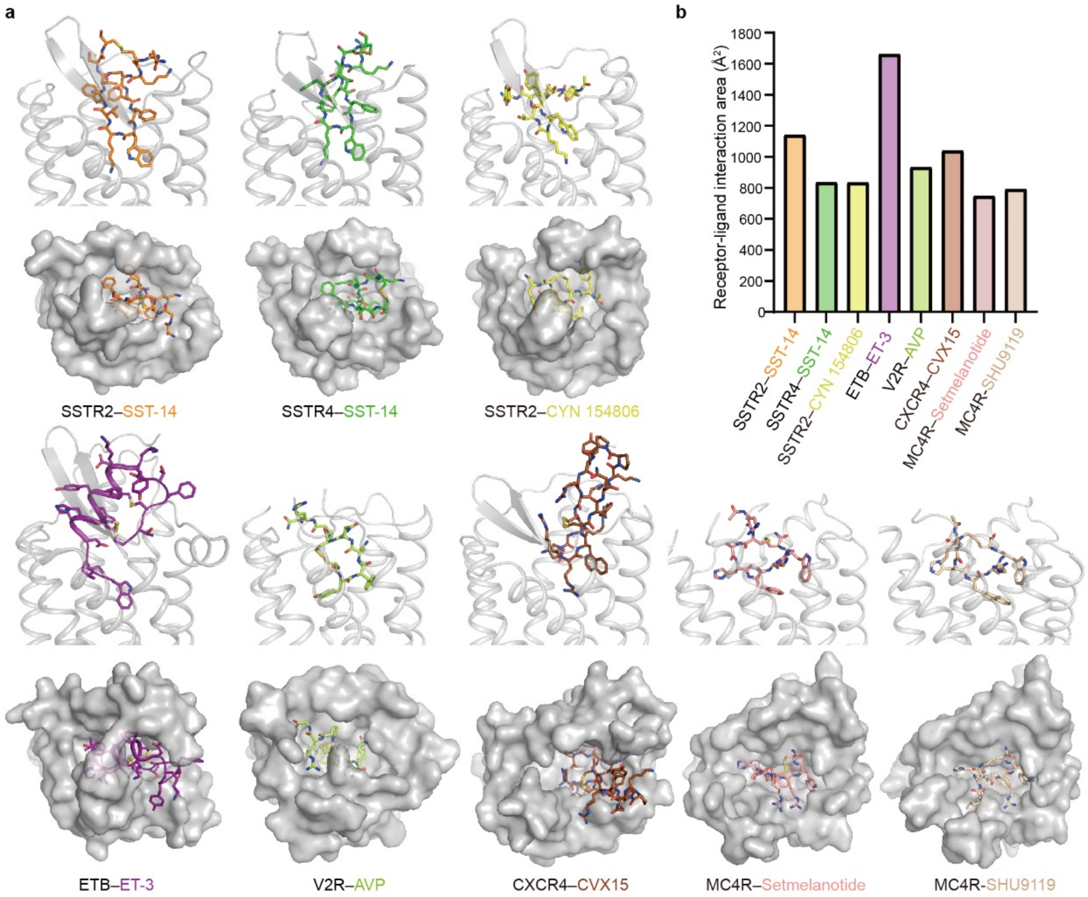

Supplementary information, Fig. S5| Ligand binding site of cyclic peptide-bound structures.

a, Ligand binding site of solved cyclic peptide-bound structures. Receptors in structures of SSTR2–SST-14, SSTR4–SST-14, SSTR2–CYN 154806, ET_B–ET-3 (PDB ID: 6IGK), V2R–AVP (PDB ID: 7KH0), CXCR4–CVX15 (PDB ID: 3OE0), MC4R–Setmelanotide (PDB ID: 7AUE), MC4R–SHU9119 (PDB ID: 6W25) are shown as cartoon and surface representation and colored by gray. The cyclic peptide ligands in structures are represented as sticks and colored by orange (SST-14 in SSTR2), green (SST-14 in SSTR4), yellow (CYN 154806), purple (ET-3), limon (AVP), brown (CVX15), salmon (Setmelanotide) and wheat (SHU9119). **b**, Histogram of receptor-ligand interaction area calculated by CCP4-PISA[57].
