# Supplementary material for: Structural insights into ligand recognition and selectivity of somatostatin receptors
Source: Cell Res. 2022 Jun 23;32(8):761–72. doi: 10.1038/s41422-022-00679-x (PMC9343605; doi:10.1038/s41422-022-00679-x)
Supplement: Supplementary file 6 — Supplementary information, Figure S6 [file 41422_2022_679_MOESM6_ESM.pdf]

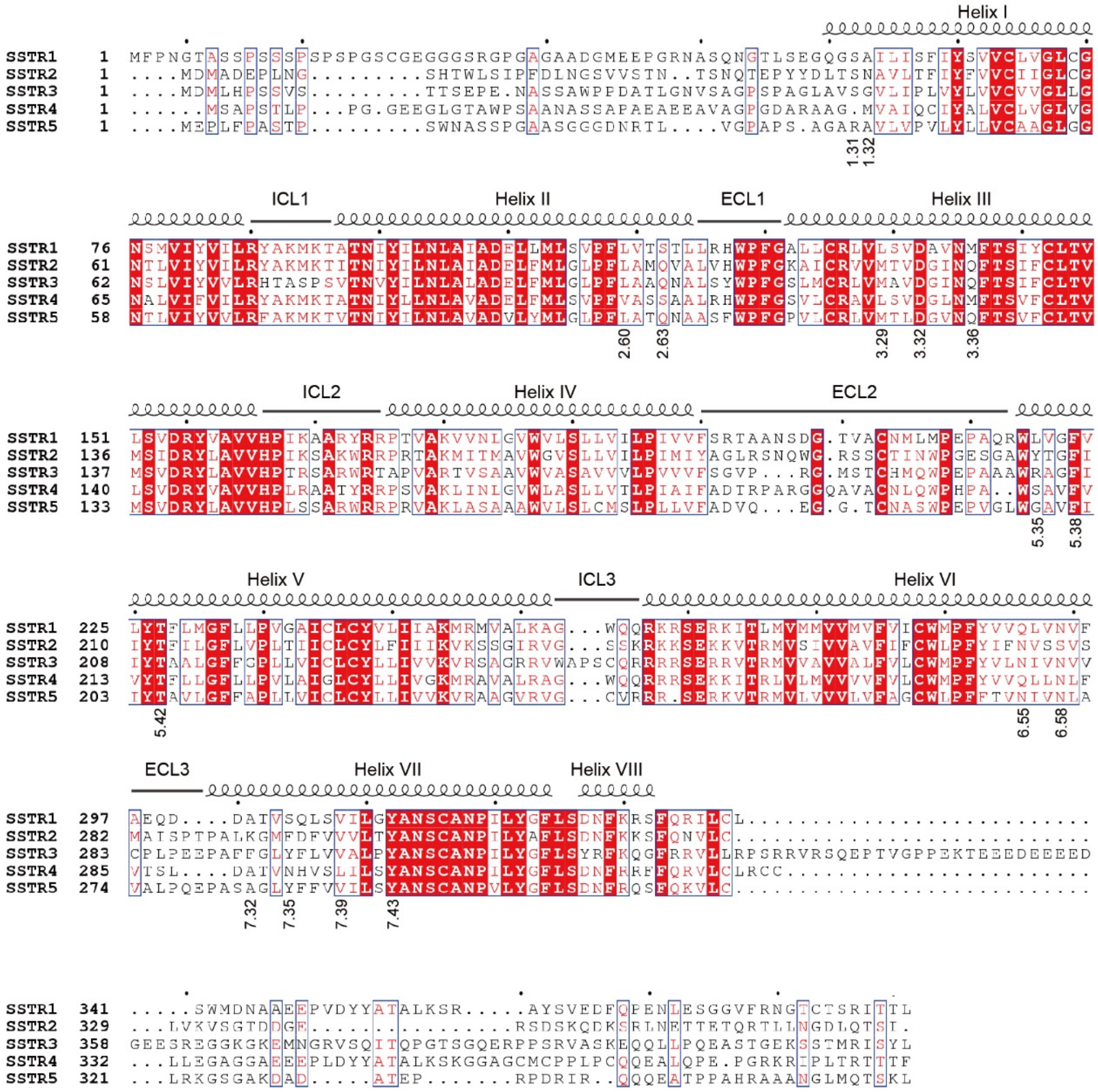

Supplementary information, Fig. S6| Sequence alignment of human SST receptors. Sequence alignment was prepared by uniport (https://www.uniprot.org/) and the graphic was generated by ESPript 3.0 server (https://espript.ibcp.fr/ESPript/). Background colors represent the degree of similarity between different receptors: red, identical; red text, similar.
