# Supplementary material for: Structural insights into ligand recognition and selectivity of somatostatin receptors
Source: Cell Res. 2022 Jun 23;32(8):761–72. doi: 10.1038/s41422-022-00679-x (PMC9343605; doi:10.1038/s41422-022-00679-x)
Supplement: Supplementary file 7 — Supplementary information, Figure S7 [file 41422_2022_679_MOESM7_ESM.pdf]

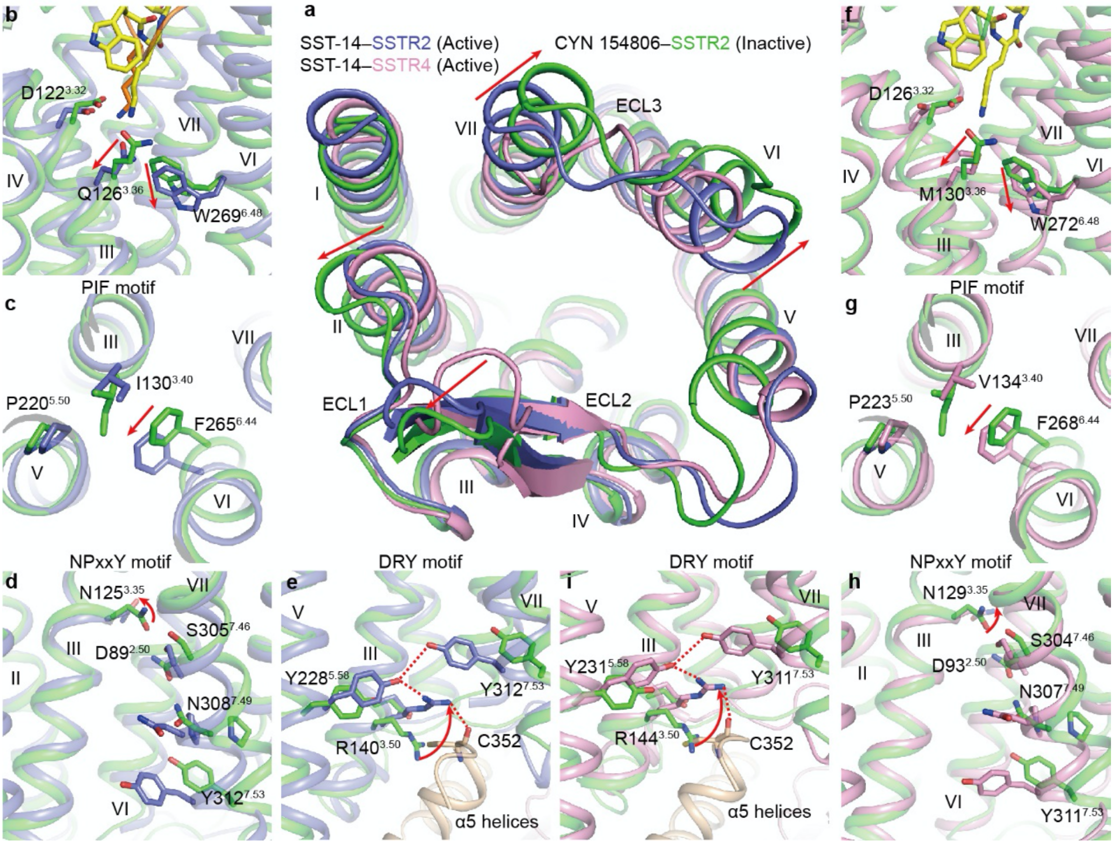

Supplementary information, Fig. S7| Activation mechanism of somatostatin receptors.

a, Structural superimposition of CYN 154806-bound SSTR2, SST-14-bound SSTR2 and SST-14-bound SSTR4. Receptors are shown as cartoon and colored by green, slate and pink respectively. Conformational changes of helices and ECL2 are indicated by red arrows. **b-e**, Dislocations of the “toggle switch”, “PI/VF” motif, “NPxxY” and “DRY” motif of SSTR2 during the receptor activation are indicated by red arrows. CYN 154806 is shown as yellow sticks, SSTR2-bound SST-14 is shown as orange sticks. Polar interactions are indicated by red dash lines. **f-i**, The dislocations of the “toggle switch”, “PI/VF” motif, “NPxxY” and “DRY” motif of SSTR4 during the receptor activation. SSTR4-bound SST-14 is shown as green cartoon.
