# Supplementary material for: Structural insights into ligand recognition and selectivity of somatostatin receptors
Source: Cell Res. 2022 Jun 23;32(8):761–72. doi: 10.1038/s41422-022-00679-x (PMC9343605; doi:10.1038/s41422-022-00679-x)
Supplement: Supplementary file 8 — Supplementary information, Figure S8 [file 41422_2022_679_MOESM8_ESM.pdf]

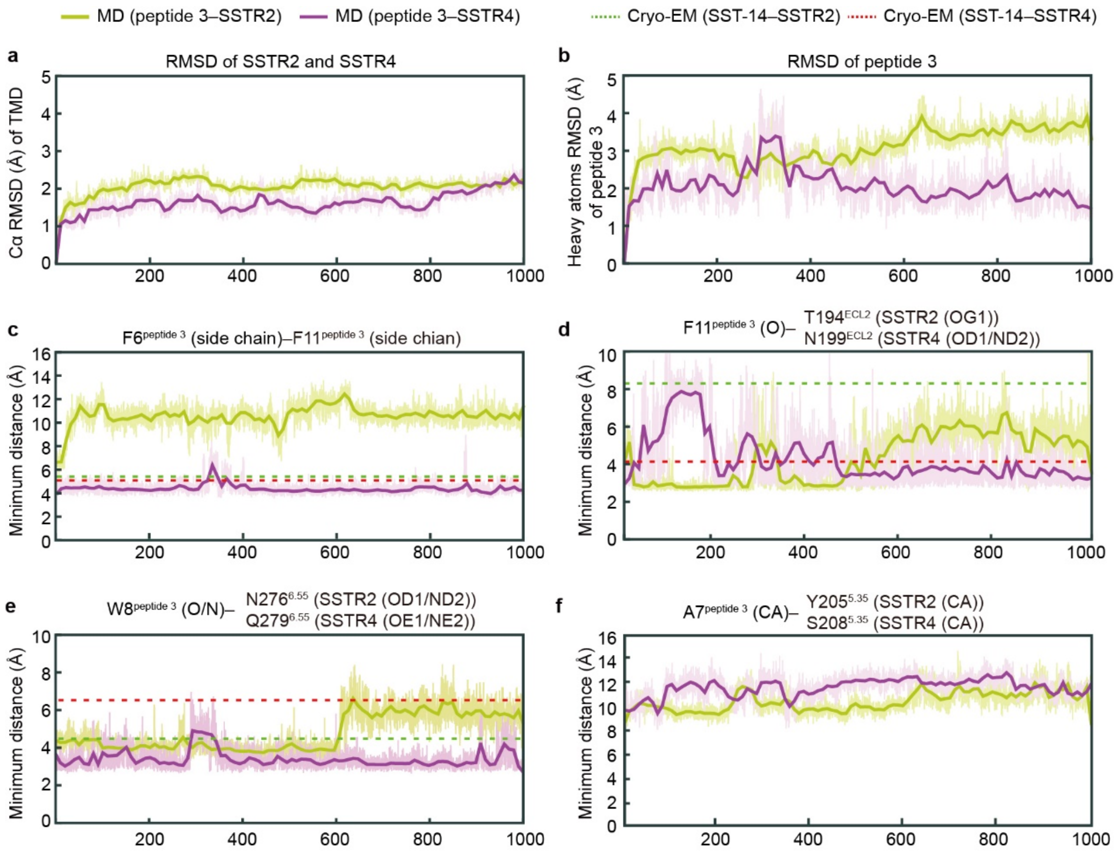


**Supplementary information Fig. S8| MD simulation of peptie 3 in SSTR2 and SSTR4**

**a**, **b**, Overall conformational changes during the molecular dynamic (MD) simulations of receptor (**a**) or peptide 3 (**b**) in complex with SSTR2 and SSTR4 in 1000 ns. Lime-green lines indicate conformational changes of receptor or peptide 3 in complex with SSTR2 during the MD simulation while ruby purple lines indicate conformational changes of receptor or peptide3 in complex with SSTR4 during the MD simulation. **c-f**, Minimum distance of three couple of interactions between receptor and ligand during the MD simulation: F6-F11, T194/N199$^{ECL2}$-F$^{11}$, N276/Q279$^{6.55}$-W$^{8}$, Y205/S208$^{5.35}$-A7.
