# Supplementary material for: Structural insights into ligand recognition and selectivity of somatostatin receptors
Source: Cell Res. 2022 Jun 23;32(8):761–72. doi: 10.1038/s41422-022-00679-x (PMC9343605; doi:10.1038/s41422-022-00679-x)
Supplement: Supplementary file 9 — Supplementary information, Figure S9 [file 41422_2022_679_MOESM9_ESM.pdf]

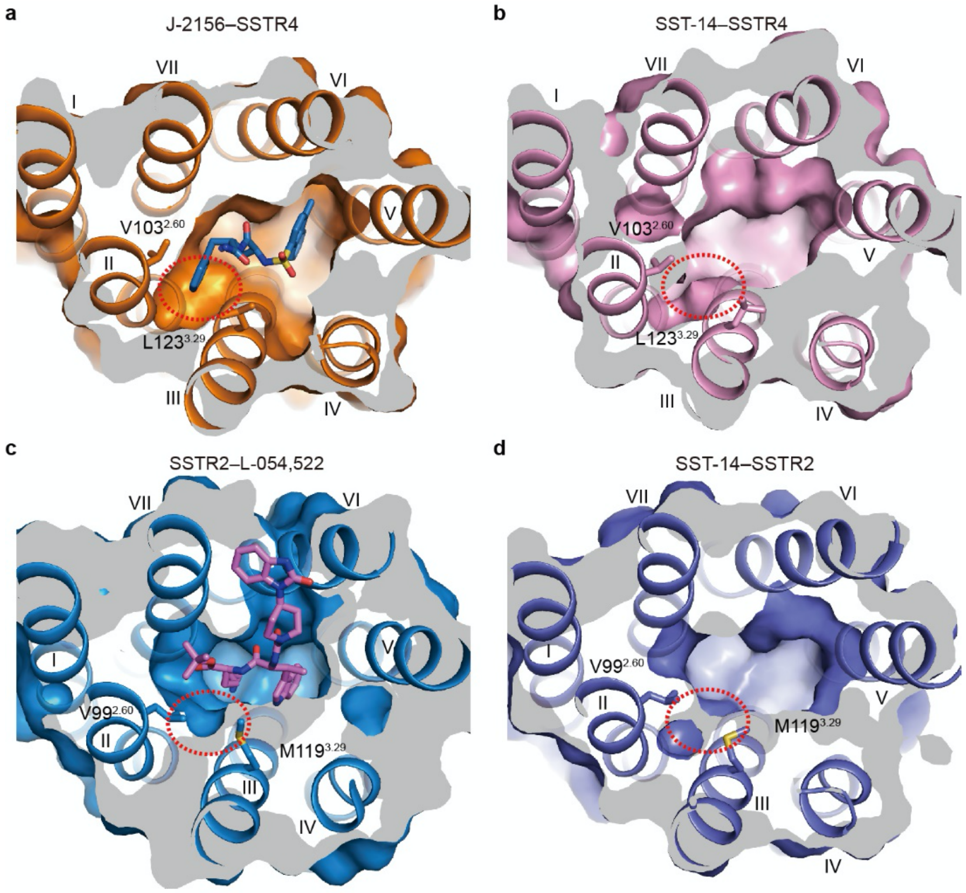

Supplementary information Fig. S9| Comparison of the sub-pocket in SSTR4.

a, **b**, Cut-view of sub-pocket of J-2156 (**a**) and SST-14-bound SSTR4 (**b**). SSTR4 is shown as orange and pink cartoon respectively. Involved residues and J-2156 are shown as sticks. Red circular dotted boxes indicate the sub-pocket formed by helices II and III. **c**, **d**, Cut-view of L-054,522 (**c**) and SST-14-bound SSTR2 (**d**). SSTR2 is shown as protactinium and slate cartoon respectively. Involved residues and L-054,522 are shown as sticks. Red circular dotted boxes indicate that the sub-pocket is disrupted by $L99^{2.60}$ and $M119^{3.29}$ in SSTR2.
