# Supplementary material for: Structural insights into ligand recognition and selectivity of somatostatin receptors
Source: Cell Res. 2022 Jun 23;32(8):761–72. doi: 10.1038/s41422-022-00679-x (PMC9343605; doi:10.1038/s41422-022-00679-x)
Supplement: Supplementary file 10 — Supplementary information, Figure S10 [file 41422_2022_679_MOESM10_ESM.pdf]

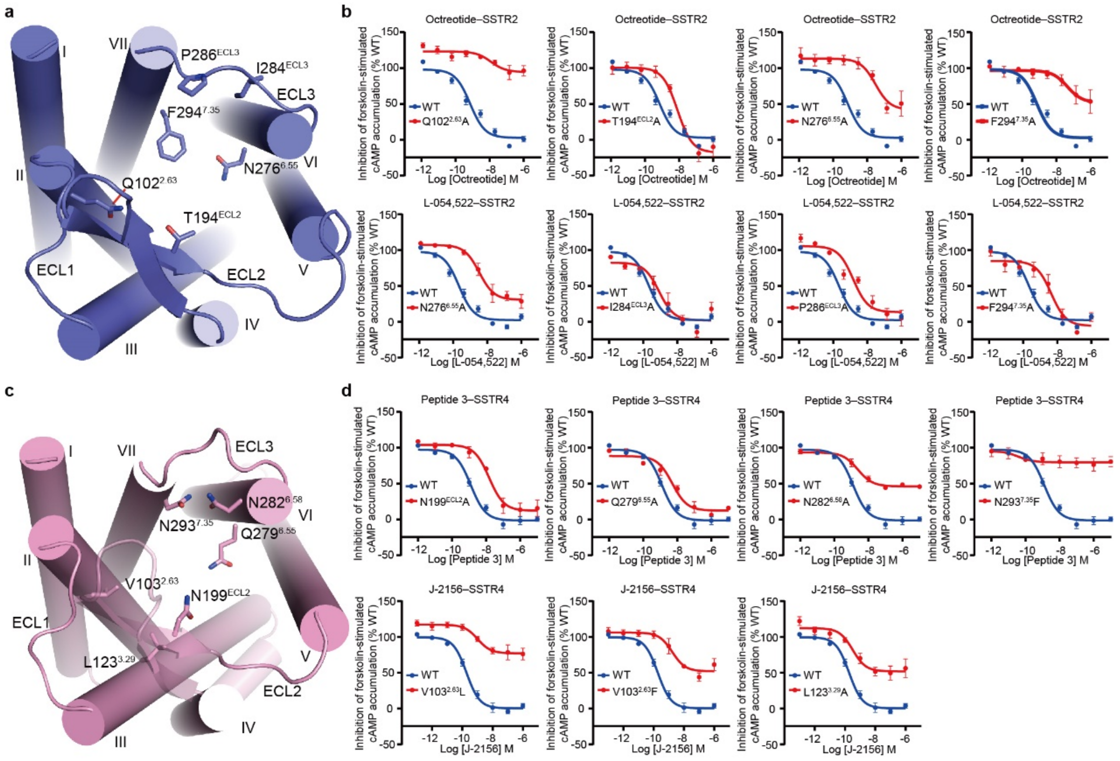

Supplementary information Fig. S10| Selectivity mechanism between SSTR2 and SSTR4.

a, Model of SSTR2 is shown as slate cylindrical helices. Residues which are critical for ligand selectivity are shown as sticks. **b**, Dose-response curves of agonist-induced inhibition of forskolin-simulated cAMP accumulation of SSTR2. All data are shown as mean ± SEM from at least three independent experiments performed in triplicate. Detailed statistical evaluation is shown in Supplementary information Table S3. **c**, Model of SSTR4 is shown as pink cylindrical helices. Residues which are critical for ligand selectivity are shown as sticks. **d**, Dose-response curves of agonist-induced inhibition of forskolin-simulated cAMP accumulation of SSTR4. All data are shown as mean ± SEM from at least three independent experiments performed in triplicate. Detailed statistical evaluation is shown in Supplementary information Table S3.
