# Supplementary material for: Structural insights into ligand recognition and selectivity of somatostatin receptors
Source: Cell Res. 2022 Jun 23;32(8):761–72. doi: 10.1038/s41422-022-00679-x (PMC9343605; doi:10.1038/s41422-022-00679-x)
Supplement: Supplementary file 11 — Supplementary information, Table S1 [file 41422_2022_679_MOESM11_ESM.pdf]

Supplementary information Table S1| X-ray data collection and refinement statistics of SSTR2–CYN 154806 and SSTR2–L-054,522 complex structures

	SSTR2–CYN 154806<br>PDB-7XNA	SSTR2–L-054,522<br>PDB-7XN9
**Data collection**		
Space group	$P2_12_12_1$	$C222_1$
Cell dimensions		
a, *b*, *c* (Å)	42.15, 85.21, 163.69	74.52, 97.96, 170.50
α, *β*, *γ* (°)	90, 90, 90	90, 90, 90
Resolution (Å)	40.82-2.65 (2.745-2.65) [a]	37.26-2.60 (2.693-2.60) [b]
CC1/2	0.954	0.996
Mean *I/σ(I)*	3.10	10.91
Completeness (%)	94.1	97.65
Redundancy	3.69	7.50
**Refinement**		
Resolution (Å)	50-2.65	50-2.60
No. reflections	17,495 (1,709)	19,455 (1,875)
R*work*/R*fre*e (%)	0.2196/0.2904	0.2356/0.2805
No. atoms		
Protein	3694	3725
Ligand/ion	84	62
Average *B*-factors (Å^2)		
Protein	58.67	64.15
Ligand	71.37	70.31
R.m.s. deviations		
Bond lengths (Å)	0.01	0.008
Bond angles (°)	1.21	0.92
Ramachandran plot (%)		
Favored	95.74	97.22
Allowed	4.26	2.78
Disallowed	0.00	0.00

[a]11 crystals were used for structure determination of SSTR2–CYN 154806 complex. Values in parentheses are for highest- resolution shell.

[b]24 crystals were used for structure determination of SSTR2–L-054,522 complex. Values in parentheses are for highest- resolution shell.
