# Supplementary material for: Structural insights into ligand recognition and selectivity of somatostatin receptors
Source: Cell Res. 2022 Jun 23;32(8):761–72. doi: 10.1038/s41422-022-00679-x (PMC9343605; doi:10.1038/s41422-022-00679-x)
Supplement: Supplementary file 12 — Supplementary information, Table S2 [file 41422_2022_679_MOESM12_ESM.pdf]

Supplementary information Table S2| Cryo-EM data collection refinement and validation statistics of the SST14–SSTR2–G_i, SST14–SSTR4–G_i and J-2156–SSTR4–G_i complex structures.

	SST-14–SSTR2–G_i PDB-7XMR	SST-14–SSTR4–G_i PDB-7XMS	J-2156–SSTR4–G_i PDB-7XMT
**Data collection and processing**			
Magnification	81,000	81,000	81,000
Voltage	300	300	300
Electron exposure (e^-/ Å^2)	70	70	70
Defocus range (μm)	-1.3 ~ -2.3	-1.3 ~ -2.3	-1.3 ~ -2.3
Pixel size (Å)	1.045	1.045	1.045
Symmetry imposed	C1	C1	C1
Initial particle projections (no.)	3,185,362	4,096,528	2,338,149
Final particle projections (no.)	696,255	799,646	600,908
Map resolution (Å)	3.1	2.9	2.8
FSC threshold	0.143	0.143	0.143
Map resolution range (Å)	2.5-5.0	2.5-5.0	2.5-5.0
**Refinement**			
Initial model used (PDB code)	6DDE, 6LML	6DDE, 6LML	6DDE, 6LML
Model resolution (Å)	3.1	2.9	2.8
FSC threshold	0.5	0.5	0.5
Map sharpening B factor (Å^2)	-118	-89	-78
Model composition			
Protein residues	871	1128	1068
Receptor residues	285	276	265
G protein residues	572	594	570
Antibody residues	/	231	233
Ligand residues	14	12	/
B factors (Å^2)			
Protein	66.71	54.86	41.81
Ligand	87.82	102.03	85.45
R.m.s. deviation			
Bond lengths (Å)	0.002	0.002	0.002
Bond angles (°)	0.452	0.466	0.502
Validation			
MolProbity score	1.46	1.68	1.54
Clashscore	7.98	8.52	7.88
Rotamer outlier (%)	0.00	0.00	0.00
Ramachandran plot			
Favored (%)	97.90	96.63	97.46
Allowed (%)	2.10	3.37	2.54
Disallowed (%)	0.00	0.00	0.00
