# Supplementary material for: Structural insights into ligand recognition and selectivity of somatostatin receptors
Source: Cell Res. 2022 Jun 23;32(8):761–72. doi: 10.1038/s41422-022-00679-x (PMC9343605; doi:10.1038/s41422-022-00679-x)
Supplement: Supplementary file 13 — Supplementary information, Table S3 [file 41422_2022_679_MOESM13_ESM.pdf]

Supplementary information Table S3| Agonists induced inhibition of forskolin-stimulated cAMP accumulation of SSTR2 and SSTR4.

SST14-induced inhibition of forskolin stimulated cAMP accumulation of SSTR2

Mutants[a]	EC$_{50}$ (nM)	EC$_{50}$ ratio[b]	pEC50± SEM[c]	Span[c,d] (% of WT)	n[e]	Expression[f] (% of WT)
WT	0.83	1	9.08±0.07	100±3	20	100
Construct 1[g]	0.96	1	9.02±0.14	119±7	3	117±16
Q102$^{2.63}$A	7.2	9	8.15±0.27***	60±6**	3	52±3***
D122$^{3.32}$A	nd	/	nd	35±21****	3	128±16
Q126$^{3.36}$A	15.3	18	7.82±0.17****	87±6	3	90±6
T194^{ECL2}A	31.2	38	7.51±0.12****	110±5	4	97±6
Y205$^{5.35}$W	5.5	7	8.26±0.15**	147±10***	3	119±2
F208$^{5.38}$A	8.3	10	8.08±0.18***	132±10*	3	74±4
T212$^{5.42}$A	13.2	16	7.88±0.13****	104±6	3	78±2
F272$^{6.51}$A	507	611	6.30±0.23****	142±16**	3	60±7**
F275$^{6.54}$A	8.9	11	8.05±0.25****	103±10	4	97±3
N276$^{6.55}$A	34.7	42	7.46±0.16****	91±6	4	52±1***
I284^{ECL3}A	7.7	9	8.11±0.22***	100±9	3	74±2
P286^{ECL3}A	10.6	13	7.98±0.16****	88±6	3	68±5*
K291$^{7.32}$A	61.7	74	7.21±0.09****	119±5	3	74±4
F294$^{7.35}$A	363	437	6.44±0.18****	121±10	3	136±1**
F294$^{7.35}$N	235	282	6.63±0.23****	78±8	3	25±9****
V298$^{7.39}$A	17.3	21	7.76±0.19****	130±10	3	77±9
Y302$^{7.43}$A	20.6	25	7.69±0.20****	83±7	3	101±3

Octreotide-induced inhibition of forskolin stimulated cAMP accumulation of SSTR2

Mutants[a]	EC$_{50}$ (nM)	EC$_{50}$ ratio[b]	pEC50± SEM[c]	Span[c,d] (% of WT)	n[e]	Expression[f] (% of WT)
WT	0.71	1	9.15±0.09	100±4	20	100
Q102$^{2.63}$A	11.9	17	7.92±0.39	30±5****	4	54±4***
D122$^{3.32}$A	nd	/	nd	16±4****	3	120±11
Q126$^{3.36}$A	2.3	3	8.63±0.16	101±7	3	87±3
S192^{ECL2}A	4.8	7	8.32±0.21*	146±13***	3	102±2
T194^{ECL2}A	8.0	11	8.10±0.13**	125±7	3	95±7
Y205$^{5.35}$W	0.08	0.1	10.09±0.24*	97±10	3	118±2
F208$^{5.38}$A	12.5	18	7.90±0.15***	128±8	3	79±8
T212$^{5.42}$A	13.9	20	7.86±0.25***	90±10	3	77±2
F272$^{6.51}$A	179	252	6.75±0.20****	134±17*	3	56±7***
F275$^{6.54}$A	0.36	0.5	9.45±0.21	110±10	4	99±1
N276$^{6.55}$A	30.2	43	7.52±0.26****	75±9	3	69±9*
I284^{ECL3}A	1.5	2	8.82±0.32	58±8**	3	70±5*
P286^{ECL3}A	1.9	3	8.71±0.27	72±8	3	79±1
K291$^{7.32}$A	6.2	9	8.21±0.18*	103±8	3	80±2
F294$^{7.35}$A	156	220	6.81±0.14****	123±10	3	137±5**

F294$^{7.35}$N	44.2	62	7.36±0.39****	47±9****	3	37±3****
V298$^{7.39}$A	8.8	12	8.06±0.08**	127±4	3	77±9
Y302$^{7.43}$A	31.5	44	7.50±0.15****	147±12***	3	123±8

L-054,522-induced inhibition of forskolin stimulated cAMP accumulation of SSTR2

Mutants[a]	EC_{50} (nM)	EC_{50} ratio[b]	pEC50± SEM[c]	Span[c,d] (% of WT)	n[e]	Expression[f] (% of WT)
Wild-type	0.22	1	9.67±0.08	100±4	16	100
Construct 2[*g]	0.53	2	9.28±0.17	76±6	3	108±15
Q102$^{2.63}$A	0.34	2	9.46±0.18	79±6	5	61±3**
D122$^{3.32}$A	42	191	7.37±0.27	41±6***	3	169±12****
Q126$^{3.36}$A	0.66	3	9.18±0.17	82±6	4	77±3
T194^{ECL2}A	0.55	3	9.26±0.23	104±10	3	91±5
Y205$^{5.35}$A	0.01	0.05	11.08±0.21****	143±18**	4	131±10*
F208$^{5.38}$A	0.46	2	9.34±0.13	81±5	3	88±4
T212$^{5.42}$A	1.2	5	8.94±0.12*	108±5	3	77±2
F272$^{6.51}$A	14.1	64	7.85±0.19****	215±18****	5	67±2*
F275$^{6.54}$A	1.2	5	8.93±0.15**	154±10****	4	103±3
N276$^{6.55}$A	3.4	15	8.47±0.15****	80±5	3	77±6
I284^{ECL3}A	0.85	4	9.07±0.22	84±7	3	69±7*
P286^{ECL3}A	1.5	7	8.83±0.18**	96±7	3	68±5*
K291$^{7.32}$A	0.96	4	9.02±0.12	113±6	3	71±1*
F294$^{7.35}$A	4.4	20	8.36±0.20****	95±8	5	104±7
V298$^{7.39}$A	2.1	10	8.68±0.16***	131±9	3	77±9
Y302$^{7.43}$A	2.2	10	8.67±0.19***	96±8	3	107±6

SST14-induced inhibition of forskolin stimulated cAMP accumulation of SSTR4

Mutants[a]	EC_{50} (nM)	EC_{50} ratio[b]	pEC50± SEM[c]	Span[c,d] (% of WT)	n[e]	Expression[f] (% of WT)
Wild-type	4.8	1	8.31±0.09	100±3	17	100
Construct 4[g]	7.4	2	8.13±0.19	111±9	3	70±3
R110^{ECL1}A	6.9	1	8.16±0.12	116±6	3	74±6
D126$^{3.32}$A	nd	/	nd	nd	3	175±12****
M130$^{3.36}$A	11.0	2	7.96±0.23	91±9	3	127±4
N199^{ECL2}A	19.8	4	7.70±0.15	125±8	3	131±21
S208$^{5.35}$A	3.7	1	8.43±0.15	99±6	3	78±10
S208$^{5.35}$W	47.3	10	7.33±0.19***	53±4****	3	13±2****
F211$^{5.38}$A	144	30	6.84±0.16****	93±7	3	101±20
T215$^{5.42}$A	8.2	2	8.09±0.19	111±9	3	59±7*
F275$^{6.51}$A	232	48	6.63±0.19****	111±10	3	61±7
Q279$^{6.55}$A	103	21	6.99±0.18****	94±8	3	66±7
N282$^{6.58}$A	47.0	10	7.33±0.12***	87±4	3	48±5**
N293$^{7.35}$F	43.3	9	7.36±0.40**	34±6****	3	15±4****
L297$^{7.39}$A	123	26	6.91±0.12****	170±9****	3	64±3
Y301$^{7.43}$A	164	34	6.79±0.14****	101±6	3	74±1

Peptide3-induced inhibition of forskolin stimulated cAMP accumulation of SSTR4

Mutants[a]	EC_{50} (nM)	EC_{50} ratio[b]	pEC50± SEM[c]	Span[c,d] (% of WT)	n[e]	Expression[f] (% of WT)
Wild-type	1.3	1	8.90±0.08	100±3	14	100
R110^{ECL1}A	1.9	1	8.72±0.14	100±6	5	75±6*
D126$^{3.32}$A	nd	nd	nd	nd	3	129±7**
M130$^{3.36}$A	98.3	76	7.01±0.12****	72±4***	5	70±4**
N199^{ECL2}A	15.0	12	7.82±0.14***	93±6	3	98±4
S208$^{5.35}$W	1.1	1	8.95±0.24	58±6****	3	13±2****
F211$^{5.38}$A	145	112	6.84±0.17***	65±5****	4	66±5**
T215$^{5.42}$A	0.48	0.4	9.32±0.19	79±6	3	77±3*
F275$^{6.51}$A	683	525	6.17±0.45****	20±5****	3	45±3***
Q279$^{6.55}$A	5.0	4	8.30±0.15	77±5*	3	82±2
N282$^{6.58}$A	2.3	2	8.63±0.13	48±2***	3	67±3**
N293$^{7.35}$A	3.5	3	8.46±0.16	106±7	3	95±13
N293$^{7.35}$F	nd	/	nd	16±7****	3	15±4****
L297$^{7.39}$A	101	78	6.99±0.18****	99±8	4	65±7***
Y301$^{7.43}$A	nd	/	nd	nd	3	85±5
**J-2156-induced inhibition of forskolin stimulated cAMP accumulation of SSTR4**						
Mutants[a]	EC_{50} (nM)	EC_{50} ratio[b]	pEC_{50}± SEM[c]	Span[c,d] (% of WT)	n[e]	Expression[f] (% of WT)
Wild-type	0.19	1	9.73±0.08	100±3	15	100
Construct 4[g]	0.03	0.2	10.46±0.13*	106±5	3	79±6
V103$^{2.60}$L	1.0	5	9.00±0.25*	40±4****	3	55±11****
L123$^{3.29}$A	0.32	2	9.49±0.23	61±6**	3	69±4**
D126$^{3.32}$A	nd	nd	nd	23±28****	3	175±12****
M130$^{3.36}$A	0.75	4	9.12±0.19*	78±6	4	74±3*
N199^{ECL2}A	0.06	0.3	10.23±0.14	88±5	3	151±3****
F211$^{5.38}$A	6.3	33	8.20±0.17****	94±7	3	65±5**
T215$^{5.42}$A	0.14	1	9.85±0.14	92±5	3	82±3
F275$^{6.51}$A	nd	nd	nd	22±7****	3	54±4****
Y276$^{6.52}$A	193	1016	6.71±0.08****	106±6	3	76±7*
Q279$^{6.55}$A	0.27	1	9.57±0.17	98±6	3	82±2
N282$^{6.58}$A	0.10	1	9.99±0.21	62±5**	3	67±3**
N293$^{7.35}$A	0.50	3	9.30±0.17	87±6	5	86±2
L297$^{7.39}$A	26.0	137	7.59±0.16****	111±8	3	60±5***
Y301$^{7.43}$A	94.5	497	7.02±019****	64±7**	4	87±5

[a]Mutants represent that mutations were introduced separately into the WT receptors and transiently expressed in HEK 293F cells.

[b]The EC_{50} ratio was shown as $EC_{50(mutant)}/EC_{50(WT)}$, indicating the shift between the WT and mutant curves, reflecting the effect of the mutations on receptor signaling.

[c]Data are shown as mean ± S.E.M. from at least three independent experiments

performed in technical triplicate. One-way ANOVA was performed followed by Dunnett's post-test and compared with WT. The P value was defined as: *P<0.05; **P<0.01; ***P<0.001; ****P<0.0001.

[d] The span is defined as the window between the maximal agonists response (E_{max}) and vehicle (no ligand).

[e] Sample size; the number of independent experiments performed in technical triplicate.

[f] Receptor expression level of HEK 293F cells was determined independently by flow cytometry with anti-FLAG antibody and shown as per cent compared to the WT.

[g] Constructs for structure determination. "Constructs 1" of SSTR2 was used for cryo-EM structure determination with SST14, containing 10 residues truncation at C terminus. "Constructs 2*" of SSTR2 was used for crystallization with L-054,522, containing 2 mutations (V106E^{ECL1}, S316$^{8.47}$D) and 10 residues truncation at C terminus with an intact ICL3. "Constructs 4" of SSTR4 was used for cryo-EM structure determination with SST14 and J-2156, containing 1 mutations (V264F$^{6.40}$) and 60 residues truncation at C terminus.
