# Supplementary material for: Structural insights into ligand recognition and selectivity of somatostatin receptors
Source: Cell Res. 2022 Jun 23;32(8):761–72. doi: 10.1038/s41422-022-00679-x (PMC9343605; doi:10.1038/s41422-022-00679-x)
Supplement: Supplementary file 14 — Supplementary information, Table S4 [file 41422_2022_679_MOESM14_ESM.pdf]

Supplementary information Table S4| Binding of ligands to wild-type (WT) and mutant SSTR2 or SSTR4 in competition with ^{125}I-Tyr11-SST14.

SST14 binding to SSTR2 in competition with ^{125}I-Tyr^{11}-SST14					
Mutants[a]	IC_{50} (nM)	IC_{50} ratio[b]	pIC50± SEM[c]	Span[c,d] (% of WT)	n[e]
WT	1.4	1	8.86±0.09	100±4	5
Construct 1[f]	7.6	5	8.12±0.39	39±6****	3
Q102$^{2.63}$A	832	594	6.08±0.53**	22±7****	3
D122$^{3.32}$A	2222	1587	5.65±0.93**	23±14****	3
Q126$^{3.36}$A	1520	1086	5.82±0.78**	21±10****	3
T194^{ECL2}A	14.6	10	7.83±1.18	14±7****	3
Y205$^{5.35}$A	1.5	1	8.82±0.20	60±5**	3
T212$^{5.42}$A	1.4	1	8.87±0.25	68±7*	3
F272$^{6.51}$A	162	116	6.79±0.54	19±5****	3
F275$^{6.54}$A	301	215	6.52±0.36*	51±8***	3
I284^{ECL3}A	3569	2549	5.45±0.61**	30±13****	3
F294$^{7.35}$A	208	149	6.68±0.50	18±4****	3
Y302$^{7.43}$A	524	374	6.28±0.71*	19±7****	3
**Octreotide binding to SSTR2 in competition with ^{125}I-Tyr^{11}-SST14**					
Mutants[a]	IC_{50} (nM)	IC_{50} ratio[b]	pIC50± SEM[c]	Span[c,d] (% of WT)	n[e]
WT	5.6	1	8.25±0.16	100±7	4
Q102$^{2.63}$A	8.1	1	8.09±0.38	27±4****	3
D122$^{3.32}$A	nd	/	nd	16±7****	3
Q126$^{3.36}$A	nd	/	nd	16±8****	3
Y205$^{5.35}$A	0.19	0.03	9.73±0.17	60±5**	3
F272$^{6.51}$A	32.9	6	7.48±0.50	26±6****	3
F275$^{6.54}$A	5.4	1	8.27±0.19	51±4***	3
F294$^{7.35}$A	31.4	6	7.50±0.72	28±9****	3
Y302$^{7.43}$A	nd	/	nd	18±7****	3
**L-054,522 binding to SSTR2 in competition with ^{125}I-Tyr^{11}-SST14**					
Mutants[a]	IC_{50} (nM)	IC_{50} ratio[b]	pIC50± SEM[c]	Span[c,d] (% of WT)	n[e]
Wild-type	2.9	1	8.53±0.13	100±6	3
Construct 2[f]	8.9	3	8.05±0.50	20±4****	3
Q102$^{2.63}$A	442	152	6.35±0.52**	20±5****	3
D122$^{3.32}$A	nd	/	nd	15±7****	3
Q126$^{3.36}$A	nd	/	nd	15±5****	3
T194^{ECL2}A	35.5	12	7.45±0.59	22±6****	3
Y205$^{5.35}$A	1.3	0.4	8.89±0.15	70±5**	3
T212$^{5.42}$A	3.2	1	8.49±0.11	94±5	3
F272$^{6.51}$A	nd	/	nd	13±3****	3
F275$^{6.54}$A	26.7	9	7.57±0.23	33±3****	3

$F294^{7.35}A$	nd	/	nd	19±2****	3
$Y302^{7.43}A$	nd	/	nd	15±2****454	3
**CYN 154806 binding to SSTR2 in competition with ^{125}I-Tyr^{11}-SST14**					
Mutants[a]	IC_{50} (nM)	IC_{50} ratio[b]	pIC50± SEM[c]	Span[c,d] (% of WT)	n[e]
Wild-type	4.4	1	8.36±0.13	100±6	4
Construct 3[f]	225	51	6.65±0.41**	30±6****	3
$S42^{1.31}A$	2.8	0.6	8.56±0.14	145±9***	3
$N43^{1.32}A$	8.1	2	8.09±0.18	96±8	3
$Q102^{2.63}A$	20.6	5	7.69±0.49	24±5****	3
$D122^{3.32}A$	nd	/	nd	20±2****	3
$Q126^{3.36}A$	134	30	6.87±0.33*	25±4****	3
$F272^{6.51}A$	23.2	5	7.63±0.63	21±6****	3
$F275^{6.54}A$	22.1	5	7.66±0.24	69±7**	4
$F294^{7.35}A$	22.0	5	7.66±0.37	36±6****	4
$D295^{7.36}A$	3.5	0.8	8.45±0.21	62±6**	3
$Y302^{7.43}A$	nd	/	nd	15±5****	3
**SST14 binding to SSTR4 in competition with ^{125}I-Tyr^{11}-SST14**					
Mutants[a]	IC_{50} (nM)	IC_{50} ratio[b]	pIC50± SEM[c]	Span[c,d] (% of WT)	n[e]
Wild-type	1.5	1	8.81±0.09	100±4	5
Construct 4[f]	2.1	1	8.67±0.27	68±7*	3
$D126^{3.32}A$	131	87	6.88±0.49*	27±6****	3
$M130^{3.36}A$	13.6	9	7.87±0.75	20±6****	3
$N199^{ECL2}A$	54.5	36	7.26±0.62	30±8****	3
$S208^{5.35}A$	0.94	0.6	9.03±0.11	133±6*	3
$T215^{5.42}A$	0.80	0.5	9.09±0.27	116±13	3
$F275^{6.51}A$	30.5	20	7.52±0.60	37±9****	3
$Q279^{6.55}A$	0.60	0.4	9.22±0.49	43±9***	3
$N293^{7.35}A$	0.58	0.4	9.24±0.14	182±11****	3
$Y301^{7.43}A$	10.5	7	7.98±0.63	29±8****	3
**Peptide3 binding to SSTR4 in competition with ^{125}I-Tyr^{11}-SST14**					
Mutants[a]	IC_{50} (nM)	IC_{50} ratio[b]	pIC50± SEM[c]	Span[c,d] (% of WT)	n[e]
Wild-type	3.8	1	8.42±0.19	100±7	5
$D126^{3.32}A$	nd	/	nd	23±8****	3
$M130^{3.36}A$	nd	/	nd	26±5***	3
$N199^{ECL2}A$	nd	/	nd	26±7***	3
$S208^{5.35}A$	0.07	0.02	10.15±0.17**	152±11**	3
$T215^{5.42}A$	0.07	0.02	10.17±0.36**	96±15	3
$F275^{6.51}A$	nd	10	nd	23±5****	3
$Q279^{6.55}A$	1.4	0.4	8.87±0.67	45±12**	3
$N293^{7.35}A$	1.3	0.3	8.89±0.23	132±12	3
$Y301^{7.43}A$	nd	/	nd	30±16***	3

J-2156 binding to SSTR4 in competition with ^{125}I-Tyr^{11}-SST14					
Mutants[a]	IC_{50} (nM)	IC_{50} ratio[b]	pIC50± SEM[c]	Span[c,d] (% of WT)	n[e]
Wild-type	0.62	1	9.21±0.14	100±6	3
Construct 4[f]	0.34	0.5	9.47±0.25	96±9	3
D126$^{3.32}$A	nd	/	nd	12±3****	3
M130$^{3.36}$A	nd	/	nd	12±4****	3
N199^{ECL2}A	nd	/	nd	13±3****	3
T215$^{5.42}$A	0.82	1	9.08±0.16	67±4***	3
F275$^{6.51}$A	nd	/	nd	11±4****	3
Y276$^{6.52}$A	nd	/	nd	9±3****	3
Q279$^{6.55}$A	1.2	2	8.93±0.38	26±4****	3
Y301$^{7.43}$A	nd	/	nd	15±5****	3

[a]Mutants represent that all mutations were introduced separately into the WT receptors and expressed in CHO K1 cells.

[b]The IC_{50} ratio was shown as $IC_{50(mutant)}/IC_{50(WT)}$, indicating the shift between the WT and mutant curves, reflecting the effect of the mutations on ligand binding.

[c]Data are shown as mean ± S.E.M. form at least three independent experiments performed in technical triplicate. One-way ANOVA was performed followed by Dunnett's post-test and compared with WT. The confident of P value was defined as: **P*<0.05; ***P*<0.01; ****P*<0.001; *****P*<0.0001.

[d] The span is defined as the window between the maximal agonists response (E_{max}) and vehicle (no ligand).

[e] Sample size; the number of independent experiments performed in technical triplicate.

[f] Constructs for structure determination. Constructs 1 of SSTR2 was used for cryo-EM structure determination with SST-14, containing 10 residues truncation at C terminus. Constructs 2 of SSTR2 was used for crystallization with L-054,522, containing 2 mutations (V106E^{ECL1}, S316$^{8.47}$D) and 10 residues truncation at C terminus with an ICL3-xylanse. Constructs 3 of SSTR2 was used for crystallization with CYN 154806, containing 2 mutations (D90$^{2.50}$N, V106E^{ECL1}, S316$^{8.47}$D) and 10 residues truncation at C terminus with an ICL3-xylanse. Constructs 4 of SSTR4 was used for cryo-EM structure determination with SST-14 and J-2156, containing 1 mutations (V264$^{6.40}$F) and 60 residues truncation at C terminus.
